# Substrate Stiffness-Driven Membrane Tension Modulates Vesicular Trafficking *via* Caveolin-1

**DOI:** 10.1021/acsnano.1c10534

**Published:** 2022-03-07

**Authors:** Dariusz Lachowski, Carlos Matellan, Sahana Gopal, Ernesto Cortes, Benjamin K. Robinson, Alberto Saiani, Aline F. Miller, Molly M. Stevens, Armando E. del Río Hernández

**Affiliations:** † Cellular and Molecular Biomechanics Laboratory, Department of Bioengineering, Imperial College London, London SW7 2AZ, United Kingdom; ‡ Department of Materials, Department of Bioengineering and Institute of Biomedical Engineering, Imperial College London, London SW7 2AZ, United Kingdom; § Department of Materials and Manchester Institute of Biotechnology, Faculty of Science and Engineering, The University of Manchester, Oxford Road, Manchester M13 9PL, United Kingdom; ∥ Department of Chemical Engineering and Manchester Institute of Biotechnology, Faculty of Science and Engineering, The University of Manchester, Oxford Road, Manchester M13 9PL, United Kingdom; ⊥ Manchester BIOGEL, Mereside, Alderley Park, Alderley Edge, Cheshire SK10 4TG, United Kingdom

**Keywords:** caveolae, membrane tension, mechanotransduction, vesicle trafficking, liver fibrosis

## Abstract

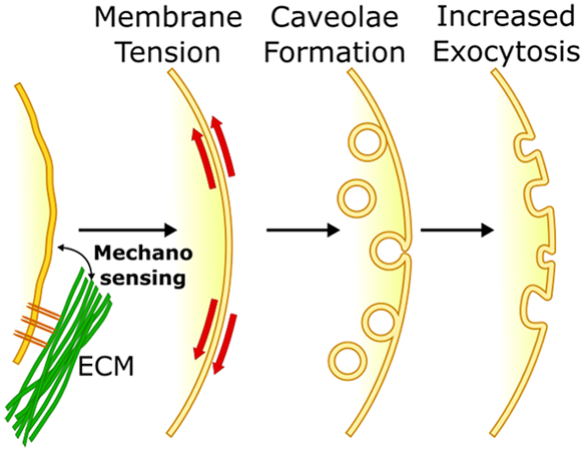

Liver fibrosis, a condition characterized by extensive deposition and cross-linking of extracellular matrix (ECM) proteins, is idiosyncratic in cases of chronic liver injury. The dysregulation of ECM remodeling by hepatic stellate cells (HSCs), the main mediators of fibrosis, results in an elevated ECM stiffness that drives the development of chronic liver disease such as cirrhosis and hepatocellular carcinoma. Tissue inhibitor of matrix metalloproteinase-1 (TIMP-1) is a key element in the regulation of ECM remodeling, which modulates the degradation and turnover of ECM components. We have previously reported that a rigid, fibrotic-like substrate can impact TIMP-1 expression at the protein level in HSCs without altering its mRNA expression. While HSCs are known to be highly susceptible to mechanical stimuli, the mechanisms through which mechanical cues regulate TIMP-1 at the post-translational level remain unclear. Here, we show a mechanism of regulation of plasma membrane tension by matrix stiffness. We found that this effect is orchestrated by the β1 integrin/RhoA axis and results in elevated exocytosis and secretion of TIMP-1 in a caveolin-1- and dynamin-2-dependent manner. We then show that TIMP-1 and caveolin-1 expression increases in cirrhosis and hepatocellular carcinoma. These conditions are associated with fibrosis, and this effect can be recapitulated in 3D fibrosis models consisting of hepatic stellate cells encapsulated in a self-assembling polypeptide hydrogel. This work positions stiffness-dependent membrane tension as a key regulator of enzyme secretion and function and a potential target for therapeutic strategies that aim at modulating ECM remodeling in chronic liver disease.

Fibrosis is a hallmark of liver diseases such as hepatocellular carcinoma,^1^ nonalcoholic fatty liver disease,^2^ hepatitis C,^3^ and other chronic liver diseases.^4^ Hepatic stellate cells (HSCs), which reside in the healthy liver in a quiescent state, become activated in response to liver injury and undergo trans-differentiation into myofibroblasts, characterized by increased contractility, excessive ECM deposition, and abnormal ECM remodeling.^5,6^ ECM remodeling is regulated by the balance between ECM deposition (fibrogenesis) and degradation. Matrix metalloproteinases (MMPs) mediate ECM turnover by degrading various ECM fibers, as well as nonfibrous components, such as adhesion proteins, matrix-associated growth factors, and inflammatory cytokines.^7−9^ MMP activity is in turn negatively regulated by tissue inhibitor of metalloproteinases (TIMPs), a structurally diverse family of proteins (TIMP-1–TIMP-4) that inhibit MMPs with different levels of affinity, thereby impairing ECM degradation.^10,11^ Dysregulation in the balance between MMPs and TIMPs results in excessive ECM deposition, reduced ECM turnover, and consequently, increased matrix stiffness, which has been found at the center of pathological conditions such as inflammation, fibrosis, and cancer.^7−9,12^ Understanding how the balance between the expression of MMPs and TIMPs in HSCs becomes disrupted in fibrosis is therefore critical in targeting the mechanical drivers of chronic liver disease.

The activity of HSCs is highly regulated by biochemical and mechanical cues from their microenvironment, and the increase in matrix stiffness associated with fibrosis has been shown to promote their activation.^13−15^ Previously, we reported that increased substrate rigidity upregulates TIMP-1 secretion by HSCs,^16^ suggesting a mechanosensitive feedback network in which the rigid environment of fibrosis can prevent its own degradation. We found that substrate stiffness has no effect on TIMP-1 mRNA expression. While this finding points toward a mechanism of stiffness-dependent regulation at the post-translational level the pathways that modulate the turnover, degradation, or transport of TIMP-1 remains largely unexplored. In cells, TIMP-1 is secreted through the cell membrane *via* exocytosis,^17^ prompting us to study the effect of matrix stiffness on membrane trafficking.

The connection between the plasma membrane, vesicular trafficking, and mechanosignaling has long been established.^18,19^ The plasma membrane is not only the physical barrier between the cell and its environment and an anchor point for a variety of mechanosensing transmembrane proteins, but also a dynamic structure that responds and adapts to both internal and external mechanical stimuli.^20^ Plasma membrane tension is sensitive to forces (*e.g.*, osmotic pressure, tension and shear) and to changes in the composition and topography (folds and invaginations) of the lipid bilayer as well as changes in the contractility of the underlying actomyosin cortex.^20−22^ While its ability to adapt to changes in tension is critical to maintain cell integrity, the lipid bilayer is itself inelastic^21^ and instead relies on changes to its surface area (*e.g.*, folds) and expansion (vesicle fusion by exocytosis) to buffer increases in tension.^20,23^

Caveolae are small (50–80 nm) invaginations of the plasma membrane rich in the transmembrane protein caveolin-1^23^ that mediate rapid and dynamic changes in plasma membrane tension in response to mechanical stimuli. The formation of caveolae occurs when exocytic caveolar carriers, rich in caveolin-1, cholesterol, and glycosphingolipids, are trafficked from the Golgi complex to the plasma membrane.^24^ The plasma membrane of cells at rest (low tension) is rich in caveolae, sometimes arranged in clusters known as rosettes,^23,25^ which serve as phospholipid reserves. In response to increased membrane tension, caveolae rapidly flatten, increasing the membrane area as a mechano-protective mechanism.^26^ Similarly, an increase in exocytosis and vesicle fusion with the membrane has also been observed as an adaptive response to high plasma membrane tension, delivering phospholipids to expand the surface area of the membrane.^21,27,28^

The mechano-adaptive role of caveolae positions them at the junction between substrate stiffness mechanosensing and membrane tension modulation. Caveolae have been shown to be closely associated with actomyosin stress fibers in several cell types,^25^ potentially *via* filamin A.^29,30^ Caveolae also flatten in response to actin polymerization,^31^ which is well-known to be positively regulated by substrate stiffness *via* mDia. Moreover, caveolin-1 expression is regulated by matrix stiffness,^32^ revealing a link between matrix mechanosensing and vesicular traffic. In turn, caveolin-1 may recruit and activate RhoA, stabilize focal adhesions, and modulate the transcription factor YAP,^23,25^ suggesting the potential for bidirectional crosstalk.

In this study, we investigate the role of substrate stiffness on plasma membrane tension and vesicular trafficking. We demonstrate that substrate stiffness positively regulates plasma membrane tension through a β1 integrin and RhoA-dependent mechanism, which in turns promotes caveolae formation and TIMP-1 vesicle exocytosis *via* caveolin-1 and dynamin-2. We then investigate the biological relevance of this mechanism by analyzing the expression of TIMP-1 and caveolin-1 in cirrhosis and hepatocellular carcinoma (HCC), and demonstrate these proteins are upregulated in chronic liver diseases, an effect that can be recapitulated on HSCs cultured on 3D matrices with fibrosis-mimicking stiffness. This work may aid in the design of therapies targeting the cell membrane to arrest the delivery of factors to the tissue microenvironment that perpetuate ECM stiffening in disease.

## Results and Discussion

### Substrate Stiffness Correlates with Plasma Membrane Tension in HSCs

Increased matrix stiffness is a hallmark of liver fibrosis and an underlying condition in many chronic liver diseases. Hepatic stellate cells (HSCs) are able to mechanically sense and respond to these signals, which increases their activation, migration, and contractility. Our previous studies showed that substrate stiffness can increase the protein levels of TIMP-1 in HSCs without affecting their mRNA expression, pointing toward an unexplored stiffness-modulated secretory mechanism.^16^ Since increased traffic of vesicles—small membrane-bound structures that transport proteins to and from the membrane for secretion—has been proposed as a response to plasma membrane tension,^33^ and plasma membrane tension can be modulated by the contractility of the actomyosin cortex,^34^ we decided to investigate the potential link between matrix stiffness and membrane tension.

**Figure 1:**
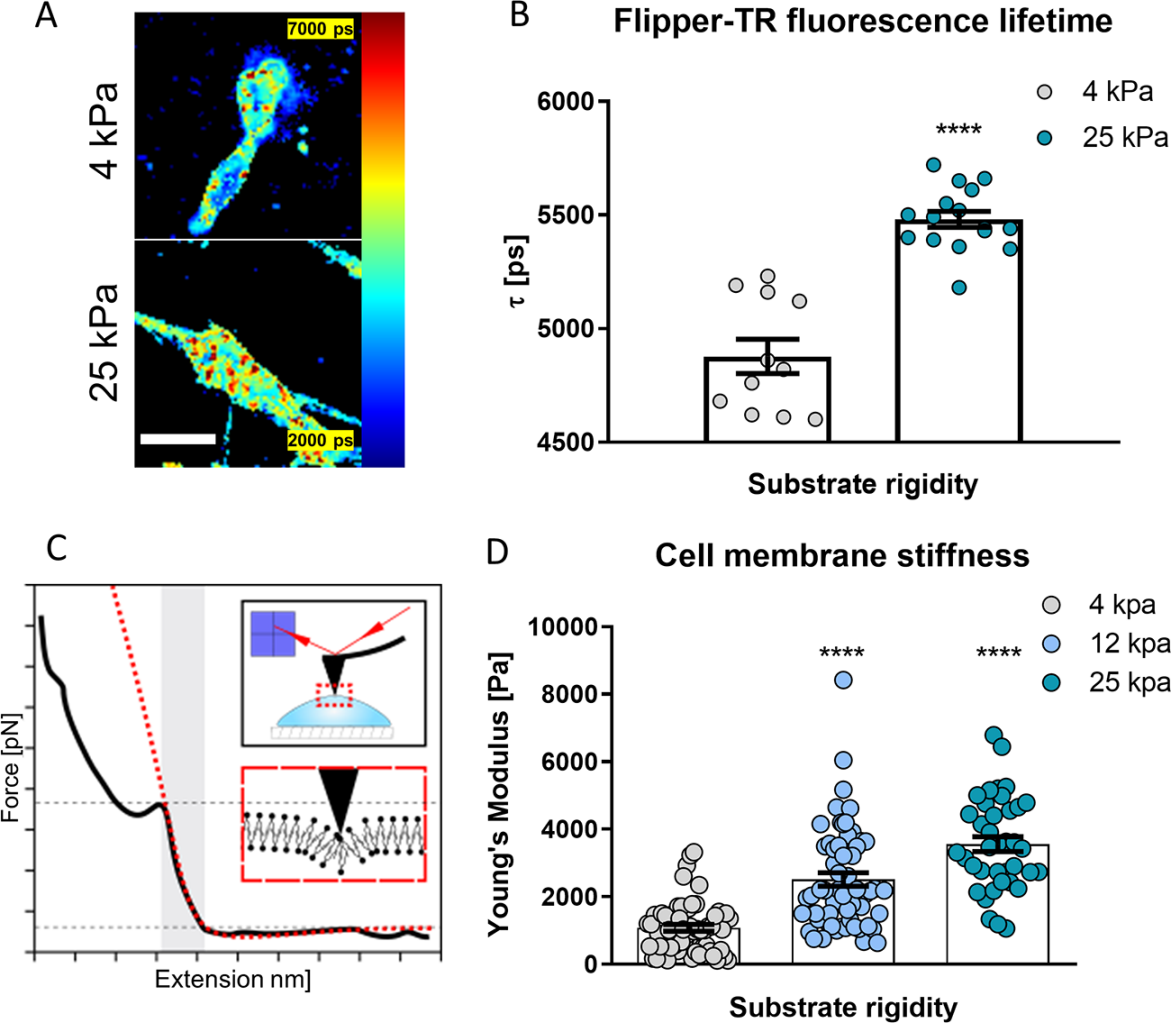
Plasma membrane tension increases in response to elevated substrate stiffness. (A) Representative images and quantification (B) of the FLIPPER-TR membrane tension probe fluorescence lifetime in hepatic stellate cells on 4 and 25 kPa polyacrylamide substrates. Scale bar represents 50 μm. Data are representative of three independent experiments; dots represent fluorescence lifetimes of *n* = 11 and 16 cells for 4 and 25 kPa substrates, respectively. Mean ± s.e.m. Markers denote the significant difference between 4 and 25 kPa by *t* test, **** *p* < 0.0001. (C) Schematics of the AFM cell membrane indentation for the Young’s Modulus measurements. (D) Hepatic stellate cell’s membrane stiffness on 4, 12, and 25 kPa polyacrylamide substrates measured as Young’s Modulus. Histogram bars represent mean ± s.e.m; dots represent individual data points. Data are representative of three independent experiments and 100 cells analyzed. Markers denote the significant difference from the 4 kPa condition by ANOVA with Dunnett’s post hoc test, **** *p* < 0.0001.

To analyze their response to substrate stiffness, HSCs were seeded on fibronectin-coated polyacrylamide gels of tunable rigidity.^35^ We then used the membrane tension reporter Flipper TR to assess the effect of substrate rigidity on membrane tension (Figure 1A,B). Flipper TR is a probe designed to intercalate within the lipid bilayer and undergo conformational changes that alter its fluorescence lifetime in response to plasma membrane tension.^36^ Using fluorescence lifetime imagining microscopy (FLIM), we observed a significant increase in the fluorescence lifetime of Flipper TR in HSCs cultured on stiff (25 kPa) polyacrylamide substrates (5481 ± 34 ps, mean ± s.e.m., *n* = 16) compared to those cultured on soft (4 kPa) substrates (4877 ± 76 ps, mean ± s.e.m., *n* = 11).

To confirm these results, we used atomic force microscopy (AFM) to probe the elasticity of the plasma membrane,^37,38^ limiting the cantilever approach velocity (2 μm/s) and the voltage set point (0.1 V) to ensure that the indentation curve corresponds to the plasma membrane and not the underlying cytoskeleton (Figure 1C). Cell membrane stiffness increased from 1.0 ± 0.1 kPa on soft (4 kPa) substrates to 3.6 ± 0.2 kPa on stiff (25 kPa) substrates (mean ± s.e.m., *n* = 49, 38 for soft and stiff respectively), indicating an increase in plasma membrane tension in response to high substrate stiffness (Figure 1D), consistent with our previous results. We also confirmed that cytoskeletal stiffness increases with substrate rigidity (Figure S1) but remains lower than membrane stiffness, consistent with a previous study.^39^ Taken together, these results indicate that HSCs are able to sense and respond to the mechanical properties of their microenvironment by modulating the plasma membrane tension, which prompted us to investigate the mechanotransduction pathway underlying this response.

### Stiffness-Driven Changes in Plasma Membrane Tension Are Dependent on the β1 Integrin/RhoA Axis

Integrins are ECM-binding transmembrane proteins that mediate the mechanical interaction between the cell and the ECM and are therefore critical in probing the mechanical properties of the microenvironment (mechanosensing) and converting these mechanical signals into biochemical signaling pathways (mechanotransduction). The fibronectin-binding β1 and β3 integrins have both been associated with matrix stiffness mechanosensing. They mediate distinct mechanotransduction pathways, namely stiffness-dependent changes in cell adhesion, and actomyosin contractility and talin-dependent mechanosignaling, respectively.^40^ Given the distinct role of β1 and β3 integrins in cell/ECM mechanical interaction and the different downstream mechanosignaling cascades, we decided to investigate which mechanosensory pathway is responsible for the changes in plasma membrane tension in response to substrate stiffness.

**Figure 2:**
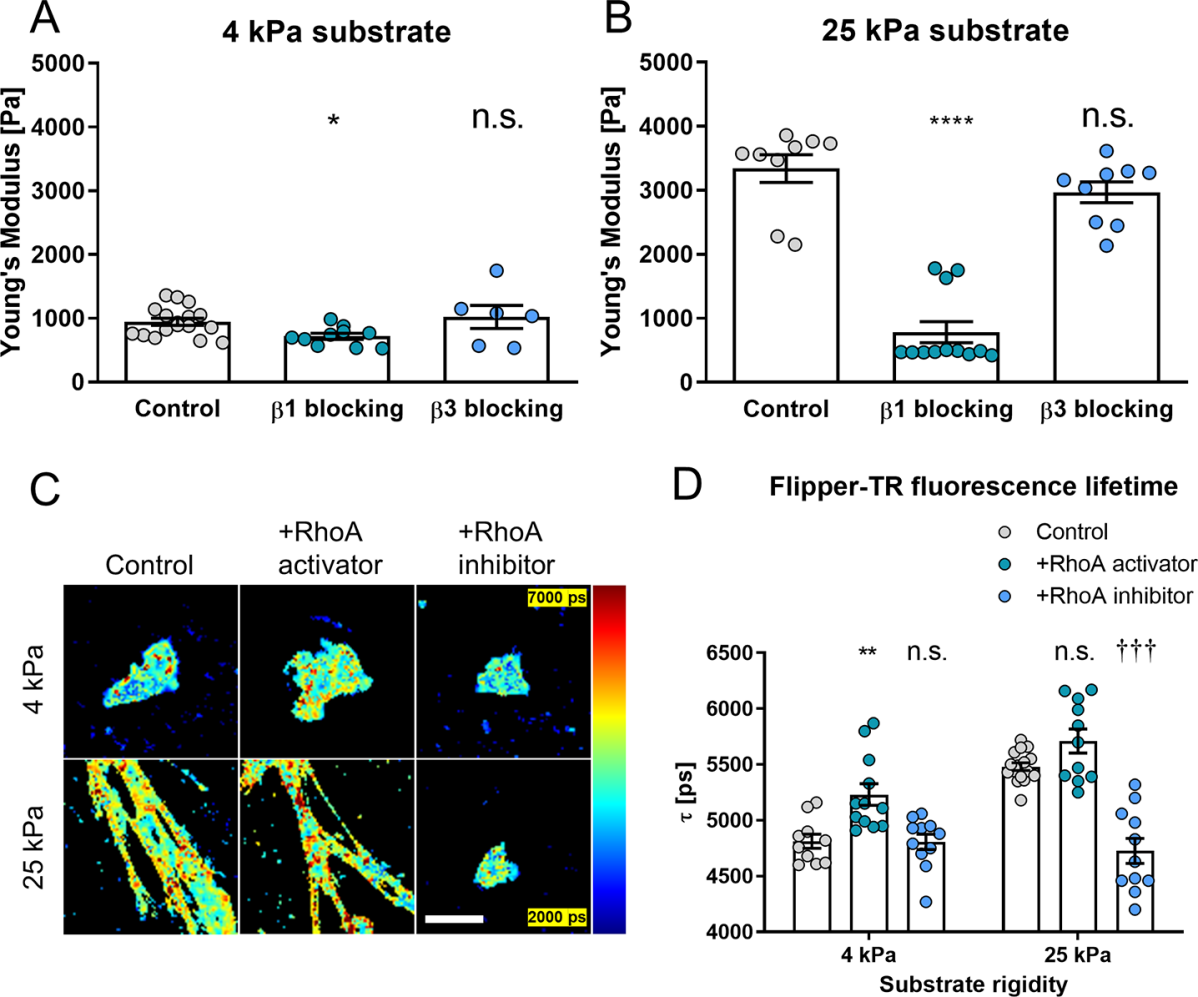
Stiffness-dependent membrane tension modulation depends on β1 integrin and RhoA. Membrane stiffness of hepatic stellate cells on 4 kPa (A) and 25 kPa (B) substrates measured by AFM. Histogram bars represent mean ± s.e.m; dots represent individual data points. Markers denote significant difference from control condition by the Kruskal–Wallis test with Dunn’s post hoc test, * 0.01 < *p* < 0.05, **** *p* < 0.0001. Representative images (C) and quantification (D) of the FLIPPER-TR membrane tension probe fluorescence lifetime (FLIM) in control hepatic stellate cells, hepatic stellate cells treated with the RhoA activator or the RhoA inhibitor on 4 and 25 kPa polyacrylamide substrates. Scale bar represents 50 μm. Data are representative of 3 independent experiments; dots represent fluorescence lifetime. Mean ± s.e.m; *n* = 10, 12, and 11 cells for control, RhoA activator and RhoA inhibitor on 4 kPa, respectively. *n* = 16, 11, and 11 cells for control, RhoA activator, and RhoA inhibitor on 25 kPa, respectively. Markers denote nonsignificant (n.s.) or significant difference from control condition on 4 kPa (*) or 25 kPa (†) by the Kruskal–Wallis test with Dunn’s post hoc test, ** 0.001 < *p* < 0.01, ††† 0.0001 < *p* < 0.001.

To this end, we used AFM to investigate the role of β1 integrin on plasma membrane tension and found that blocking of β1 integrin (552828, BD Biosciences antibody) causes a small but significant decrease in plasma membrane tension in cells cultured on soft (4 kPa) gels and, more notably, on cells cultured on stiff gels (Figure 2A,B). In particular, cells cultured on 25 kPa show a 3-fold reduction in plasma membrane tension, from 3.3 ± 0.6 kPa to 0.8 ± 0.1 kPa (mean ± s.e.m., *n* = 9 and 12, respectively) upon β1 integrin blocking, comparable to control cells on soft (4 kPa) gels. To confirm these results, we assessed mouse epithelioid cells (GE11)^41^ and observed that GE11 β1 integrin^–/–^ cells present significantly lower membrane tension compared to GE11 cells overexpressing β1 integrin (Figure S2). Together, these results suggest that the increase in plasma membrane tension we observe in response to substrate rigidity relies on β1 integrin-dependent mechanosensing.

Conversely, blocking of β3 integrin (MAB1976Z, Millipore antibody) had no significant effect on plasma membrane tension compared to control cells (Figure 2A,B), indicating that β3 integrin is dispensable for this mechanism of membrane tension regulation. These results are consistent with previous reports on the differential functions of β1 and β3 integrins in cells adhesion and mechanosensing^40^ and point toward a β1-dependent mechanotransduction pathway.

The small Rho-GTPase RhoA is an important molecular switch central to cellular mechanotransduction. β1 integrin has been shown to regulate RhoA activity *via* focal adhesion kinase (FAK) and phosphatidylinositol 3-kinase (PI3K), with integrin clustering and substrate rigidity sensing leading to an increase in RhoA activation.^42−44^ In turn, RhoA orchestrates the cell’s mechanical activity by controlling both actomyosin contractility and F-actin polymerization *via* its downstream effectors, ROCK and mDia, respectively. The β1 integrin/RhoA/ROCK pathway has been previously associated with changes in membrane structure and tension *via* RhoA/ROCK-dependent actomyosin contractility.^45^

Here, we decided to assess the role of RhoA in the substrate rigidity-dependent regulation of plasma membrane tension using used Flipper TR and FLIM. On cells treated with the RhoA activator (Rho Activator I CN01-A) we observed a significant increase in Flipper TR fluorescence lifetime (Figure 2C,D) compared to control cells—indicative of increased membrane tension. Conversely, treatment with the RhoA inhibitor (Rho Inhibitor I CT04-A) had no significant effect on the plasma membrane tension of cells cultured on 4 kPa gels but resulted in a significant decrease in fluorescence lifetime in cells cultured on 25 kPa gels. These results position the β1 integrin/RhoA axis as a key mediator between substrate rigidity and plasma membrane tension.

### Substrate Rigidity and Membrane Tension Regulate Caveolae formation

Since membrane tension increases with matrix stiffness, we hypothesized the existence of a negative feedback loop mechanism to alleviate membrane tension in response to matrix stiffness sensing. Caveolae are flasklike invaginations of the plasma membrane formed by caveolin-1 (CAV-1), an ubiquitous scaffolding and structural protein associated with the plasma membrane, the Golgi apparatus, and Golgi-derived transport vesicles^46^ that regulates vesicular transport. Caveolae flattening is a rapid (<5 min) mechanism that increases membrane surface area in response to membrane tension, which has been observed in HeLa cells, with regulation of endocytosis and exocytosis suggested as longer term regulators.^26^ Increased caveolin-1 activity has also been previously reported in response to external stiffness, promoting intracellular signaling pathways such as β1 integrin internalization^47^ and YAP nuclear translocation.^32^ These studies suggest the possibility of caveolin-1 mediated caveolae trafficking as a key response to matrix stiffness and membrane tension. As such, we decided to analyze the presence of caveolae on cell membranes under different substrate stiffness conditions.

**Figure 3:**
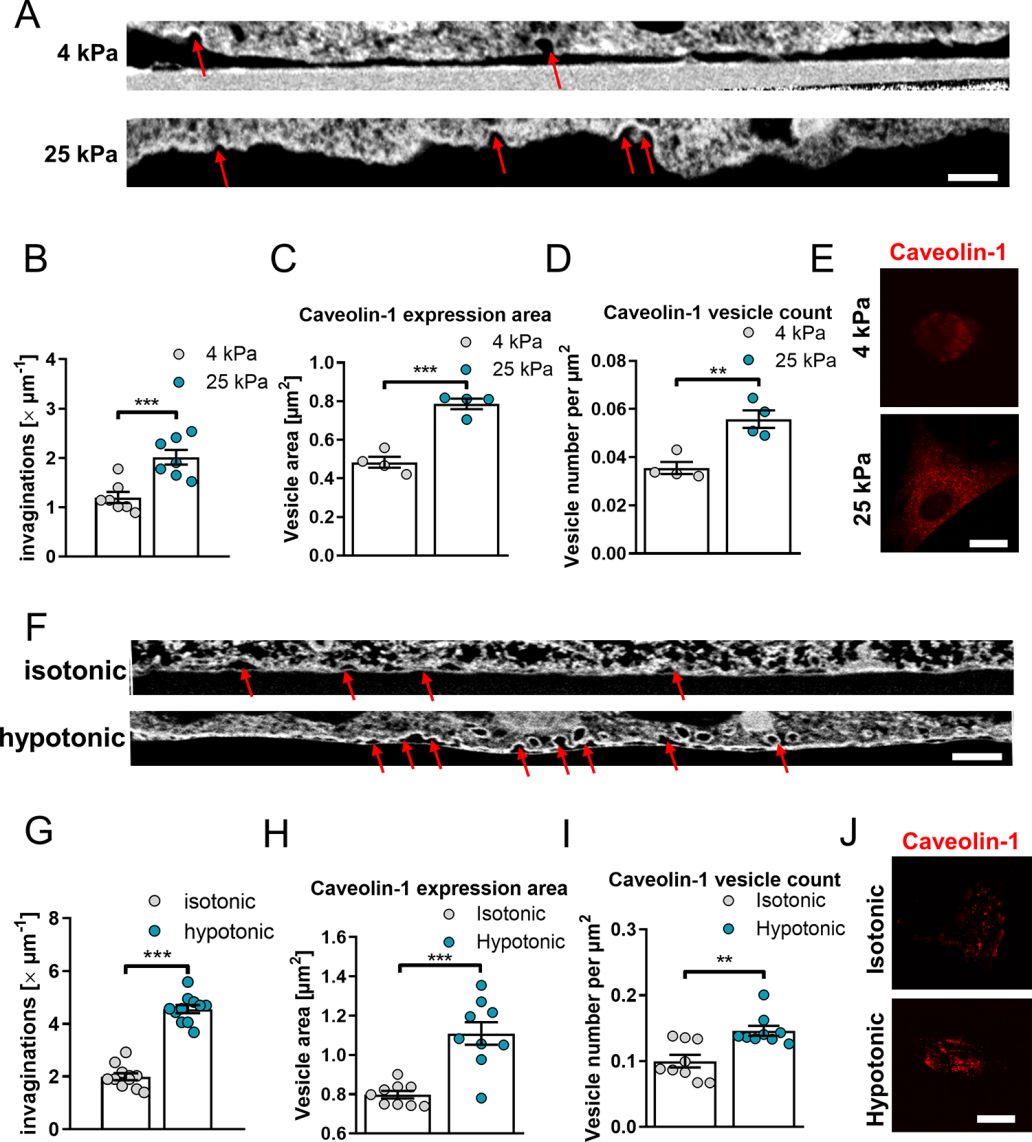
Substrate stiffness and media osmotic concentration affect caveolae formation in hepatic stellate cells. (A) FIB SEM images of the plasma membrane cross sections, red arrows mark caveolae, and (B) quantification of the number of invaginations per μm of membrane cross-section for the cells on 4 and 25 kPa polyacrylamide substrate. (C) Caveolin-1 expression area and (D) caveolae count per μm^2^ for the cells on 4 and 25 kPa polyacrylamide substrates represented on (E) caveolin-1 mCherry TIRF microscopy images. (F) FIB SEM images of the plasma membrane cross sections, red arrows mark caveolae, and (G) quantification of the number of invaginations per μm of membrane cross-section for the cells subjected to isotonic and hypotonic media. (H) Caveolin-1 expression area and (I) caveolae count per μm^2^ for the cells in isotonic and hypotonic media represented on (J) Caveolin-1 mCherry TIRF microscopy images. Scale bar in (A) and (F) represents 200 nm; scale bar in (E) and (J) represents 50 μm. Histogram bars represent mean ± s.e.m; dots represent individual data points. Data are representative of seven sections from three cells for each condition in (B) and 11 sections from three cells for each condition in (G). Four experimental replicates for (C) and (D). Eleven experimental replicates for (H) and (I). Ten cells were analyzed for each replicate in (C), (D), (H), and (I). Markers denote significant difference between groups by *t* test, ** 0.001 < *p* < 0.01, *** 0.0001 < *p* < 0.001.

To count the number of caveolae present on cell membranes, we used focused ion beam scanning electron microscopy (FIB SEM) (Figure 3A), a technique that used an ion beam to section the cell, enabling imaging of the internal microstructure in the vicinity of the membrane. For slices of each cell, we counted the total number of visible invaginations and normalized these values for the length of the slice (Figure 3B). We observed that the presence of invaginations nearly doubled on HSCs cultured on stiff (25 kPa) gels (around 2 per micron) compared to HSCs on soft (4 kPa gels) gel (around 1 per micron). We also found a slight increase in the diameter of these invaginations on HSCs cultured on stiff gels compared to those on soft gels (Supplementary Figure S3).

For further investigation, we stained caveolin-1 and measured the average vesicle size and density for cells on 4 and 25 kPa gels. (Figure 3C–E) We observed that on softer gels vesicles were significantly smaller (0.48 ± 0.03 μm^2^) compared to cells on stiffer gels (0.79 ± 0.03 μm^2^, mean ± s.e.m., *n* = 4) (Figure 3C) and in lower quantity. Similarly, vesicle density increased from ∼0.036 μm^–2^ on soft gels to ∼0.055 μm^–2^ on stiff gels (Figure 3D).

To determine the specific contribution of membrane tension and rule out substrate stiffness effects that occur independently, we altered the tonicity of the solution. A hypotonic solution has a lower concentration of solutes than an isotonic solution, leading to osmosis of water into cells and an increased membrane tension. The use of hypotonic media is an established approach for increasing membrane tension in vitro.^48^

Using FIB SEM (Figure 3F), we observed that the number of invaginations present on cell membranes under hypotonic media (150 mOsm), *i.e.*, high membrane tension, increases more than 2-fold compared to cells in isotonic media, from around 2 to around 4.5 invaginations per micron (Figure 3G). Likewise, vesicle area and density were also higher in hypotonic media (Figure 3H-J) as analyzed by total internal reflection fluorescence (TIRF) microscopy. TIRF is an imaging technique that uses a high angle of incidence to selectively illuminate a thin (∼100 nm) layer near the cell surface, enabling the visualization of vesicles close to the membrane while eliminating background signal. This indicates that membrane tension can independently promote caveolae formation and is likely to act downstream of matrix stiffness mechanosensing by β1 integrin/RhoA.

### Plasma Membrane Tension Regulates Exocytosis

We have previously shown that substrate stiffness in 2D can upregulate the expression of TIMP-1 in hepatic stellate cells (HSCs), the main effectors of liver fibrosis,^16^ thereby negatively regulating ECM remodeling and contributing to the development of fibrosis. However, this stiffness-dependent regulation was not associated with changes in TIMP-1 mRNA expression,^16^ prompting us to investigate the mechanisms that regulate its secretion. It has been previous reported that TIMP-1 can be secreted *via* vesicles incorporated into the plasma membrane during exocytosis,^17^ and increased membrane tension has been previously demonstrated to promote exocytosis in many cell lines; *e.g.*, on fibroblasts it promotes MMP secretion,^49^ while matrix stiffness has also been linked with the regulation of clathrin-mediated endocytosis.^50^ We hypothesize that the stiffness-dependent regulation of TIMP-1^16^ is mediated by a membrane tension-driven increase in vesicular trafficking and exocytosis rate.

Our previous results demonstrate that substrate stiffness and the associated increase in plasma membrane tension regulate caveolae formation. However, caveolin-1 and caveolae have been shown to participate in endocytosis, exocytosis, and transcytosis.^51,24^ To determine whether the increased presence of caveolae at the cell membrane was associated with a positive or negative feedback loop for membrane tension regulation, and to assess its potential role in stiffness-dependent TIMP-1 regulation, we set out to investigate the effects of membrane tension on the rate of exocytosis of TIMP-1.

**Figure 4:**
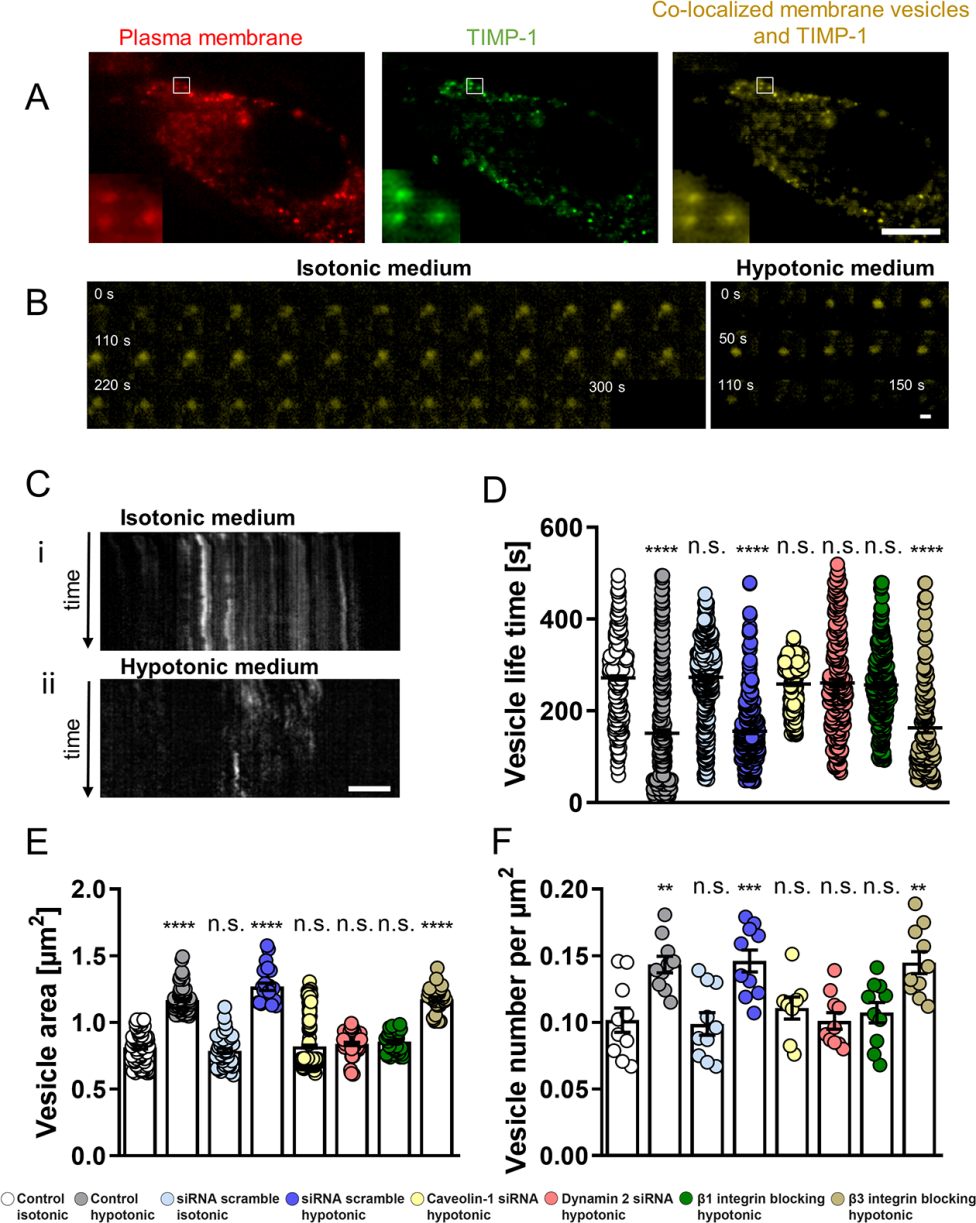
Hypotonic medium-induced plasma membrane tension affects TIMP-1 trafficking in hepatic stellate cells (HSCs). (A) TIRF images of HSCs on glass transfected with CellLight Plasma Membrane-RFP, TIMP-1 GFP, and colocalized vesicles containing TIMP-1 for exocytosis analysis. (B) Representative TIRF images for TIMP-1 containing vesicles for isotonic and hypotonic medium used for quantification of TIMP-1 vesicle lifetime. Each individual panel in (B) represents 10 s. (C) Representative TIRF time-lapse kymographs of HSC TIMP-1 vesicles in (i) isotonic and (ii) hypotonic conditions. TIMP-1 (D) vesicle lifetime, (E) vesicle area, and (F) vesicle number per μm^2^ for the cells in control isotonic, control hypotonic, siRNA scramble isotonic, siRNA scramble hypotonic, caveolin-1 siRNA hypotonic, β1 integrin blocking hypotonic, β3 integrin blocking hypotonic, and dynamin-2 siRNA hypotonic conditions. Scale bars represent (A) 20 μm, (B) 500 nm, and (C) 10 μm. Scatter dot and histogram bars represent mean ± s.e.m; dots represent individual vesicles (D, E) or cells (F). Data are representative of *n* = 3 experimental replicates. Markers denote significant difference from control isotonic condition by one way ANOVA with Dunnett’s post hoc test, ** 0.001 < *p* < 0.01, *** 0.0001 < *p* < 0.001, **** *p* < 0.0001.

To this end, we labeled both the RFP plasma membrane (red) and the intracellularly produced GFP TIMP-1 (green) in HSCs and identified TIMP-1 positive vesicles as those where the two fluorophores colocalized (Figure 4A and Figure S4). We then tracked these TIMP-1 positive vesicles over their lifetime (1 frame per 10 s) in cells cultured in isotonic or hypotonic media to determine the overall rate of exocytosis (Figure 4B) and quantified their lifetime. We observed that in hypotonic media, where membrane tension is increased, the average vesicle lifetime (151 ± 8 s) was significantly shorter than in isotonic media (272 ± 4 s, mean ± s.e.m., *n* = 281, 403 for hypotonic and isotonic, respectively), indicating that a faster rate of exocytosis correlates with membrane tension (Figure 4D).

The exocytosis rate was further visualized in a kymograph (Figure 4C), where each horizontal line of pixels represents the cell membrane at a different time point and time progresses vertically down. Slow exocytosis vesicles produce long tracks, whereas faster exocytosis vesicles produce shorter tracks. We also quantified vesicle area and count (normalized by area) for TIMP-1 vesicles in hypotonic and isotonic media (Figure 4E,F) and observed a similar trend, with cells displaying larger and more numerous vesicles in hypotonic media (high membrane tension) compared to those in isotonic media (low membrane tension).

To ensure that the increase in exocytosis was not due to an increase in intracellular TIMP-1 production, we analyzed the expression of TIMP-1 mRNA with qPCR. We observed that mRNA expression was the same for both isotonic and hypotonic media (Figure S5), indicating that membrane tension, and not TIMP-1 expression, underlies the increase in exocytosis rate.

### Caveolin-1 and Dynamin-2 Are Required for Plasma Membrane Tension-Dependent Regulation of TIMP-1 Exocytosis

To gain a mechanistic insight into the membrane tension-driven increased rate of exocytosis, we investigated caveolin-1 and dynamin-2, two proteins linked with membrane mechanics and vesicular trafficking.^23,52^ Caveolin-1 belongs to the caveolins family—proteins embedded in the cytosolic leaflet of cell membranes, with both N and C termini residing in the cytosol. They take part in protein exocytosis by first being inserted into the membrane of endoplasmic reticulum, encasing the secreted protein. They then form oligomeric complexes in the Golgi apparatus and subsequently deliver the cargo through the plasma membrane.

To elucidate the role of caveolin-1 in the regulation of TIMP-1 exocytosis, we knocked down caveolin-1 using siRNA in HSCs (Figure S6) and subjected them to hypotonic media. Using TIRF imaging (Figure 4), HSCs with caveolin-1 knockdown (siRNA) presented no difference in the TIMP-1 secretory vesicle lifetime compared to control cells in isotonic media (Figure 4D), indicating that TIMP-1 exocytosis is not responsive to membrane tension in the absence of caveolin-1. Similarly, the secretory vesicle size (Figure 4E) and count (Figure 4F) for HSCs treated with siRNA against caveolin-1 remained similar in hypotonic media compared to control cells in isotonic media. Taken together, these results indicate that caveolin-1 is required for the modulation of TIMP-1 exocytosis by membrane tension and is therefore a key component of the mechanisms of matrix stiffness-dependent TIMP-1 regulation.

Next, we turned our attention to dynamin-2, another key protein in vesicle trafficking and secretion. Of the three known isoforms of dynamin, dynamin-2 is ubiquitously expressed, while dynamins-1 and -3 are differentially expressed in brain, lungs, and testis.^52^ While dynamin-2 has been widely associated with endocytosis due to its role in vesicle neck constriction and membrane fission, it also participates in exocytosis, membrane fusion, and trans Golgi network (TGN) vesicle formation.^52−54^ In particular, dynamin-2 mediates kiss-and-run exocytosis, a method of exocytosis in which vesicle fusion with the plasma membrane is transient.^54^ Similar to caveolin-1, siRNA was used to knock down dynamin-2, and its effect on exocytosis was analyzed. Using TIRF imaging, we observed that dynamin-2 knockdown abrogates the upregulation of TIMP-1 exocytosis by hypotonic media (high membrane tension), resulting in similar vesicle lifetime, size, and count compared to control cells on isotonic media (Figure 4D–F). These results position dynamin-2 as a key element of the pathway for membrane tension-dependent regulation of vesicle trafficking along with caveolin-1.

Consistent with our previous results, β1 integrin blocking was found to inhibit the effect of hypotonic media on TIMP-1 exocytosis rate, resulting in reduced vesicle lifetime, size, and count, with levels similar to control HSCs on isotonic media (Figure 4D–F). Conversely, blocking of β3 integrin had no significant impact on the vesicle lifetime, size, or count, indicating an exocytosis rate comparable to control HSCs on hypotonic media. These findings indicate that β1 integrin mechanosensing is not only required for the substrate stiffness-driven changes in plasma membrane tension, but also for the effect of the latter on membrane trafficking and exocytosis. Moreover, we observed no changes in the expression of caveolin-1 in HSCs on hypotonic media subjected to dynamin-2 knockdown or β1 or β3 integrin blocking compared to HSCs on isotonic media (Figure S6), confirming that the observed differences in TIMP-1 secretion originate from changes in plasma membrane tension.

### TIMP-1 and Caveolin-1 Are Upregulated in Cirrhosis and Hepatocellular Carcinoma

Dysregulation of wound healing in the liver can lead to fibrosis, a condition associated with most chronic liver diseases and characterized by excessive ECM deposition and increase matrix stiffness. Abnormal matrix remodeling in fibrosis can be attributed to an imbalance between matrix metalloproteinases (MMPs), which degrade ECM fibers, and their inhibitors (TIMPs).^10,11^ Our findings here demonstrate that high substrate stiffness can increase TIMP-1 exocytosis in HSCs (the main contributors to liver fibrosis) in a caveolin-1 dependent manner through the regulation of the plasma membrane tension. We hypothesize that this mechanistic link between matrix stiffness and vesicular trafficking could be a key driver in the development of fibrosis and HCC by creating a feedback mechanism in which high matrix stiffness inhibits its own degradation *via* TIMP-1.

**Figure 5:**
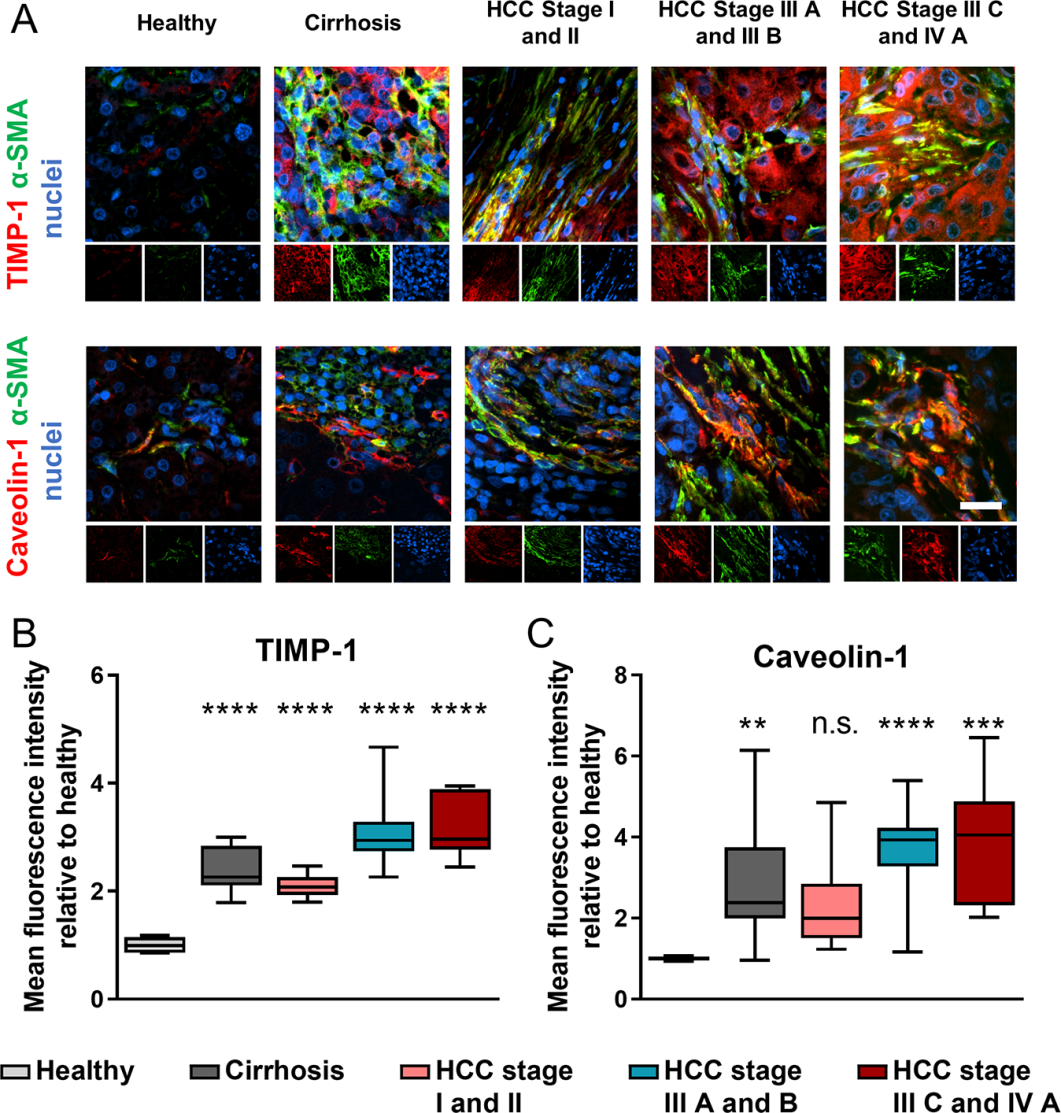
Caveolin-1 and TIMP-1 are upregulated in cirrhosis and HCC. (A) Representative epifluorescent images of human tissue caveolin-1 and TIMP-1 immunostaining of healthy, cirrhotic, and HCC (hepatocellular carcinoma) liver; protein of interest (red), alpha smooth muscle actin (green) and nuclei (blue); scale bar is 20 μm. Quantification of TIMP-1 (B), and caveolin-1 (C) immunofluorescence staining for panel (A) (*n* = number of patients: 5 healthy, 21 cirrhosis, 19 HCC stage I and II, 26 HCC stage III A and B, 7 HCC stage III C and IV A). Whiskers on box and whiskers graphs represent minimum and maximum. Markers denote significant difference from healthy condition by one-way ANOVA with Dunnett’s post hoc test, n.s.: not significant, ** 0.001 < *p* < 0.01, *** 0.0001 < *p* < 0.001, **** *p* < 0.0001.

To investigate whether this mechanism of stiffness-dependent vesicular trafficking modulation is associated with the development of chronic liver disease, we decided to assess the differential expression of caveolin-1 and TIMP-1 in liver tissues in health and disease. To this end, immunofluorescence was used to analyze their expression in tissue microarrays (TMAs) containing liver tissue samples derived from healthy donors as well as patients with cirrhosis or hepatocellular carcinoma (HCC) at various stages (Figure 5A). Simultaneously, we identified activated HSCs (myofibroblasts) in the tissue microarrays as α-smooth muscle actin (α-SMA) positive cells, a widespread marker for activated HSCs. Activated HSCs are responsible for matrix remodeling and tumor desmoplasia, a key driver in the progression of HCC.

In healthy tissue samples, we found low expression of either TIMP-1 or caveolin-1 as well as negligible presence of activated HSCs. Conversely, cirrhotic tissues showed a 2-fold increase in the expression of TIMP-1 and a 3-fold increase in the expression of caveolin-1 relative to healthy tissues as well as a marked abundance of activated HSCs (Figure 5B,C). In HCC tissue samples, the increase in TIMP-1 and caveolin-1 expression was dependent on the stage of the tumor, with early stages showing a 2-fold increase in TIMP-1 and no significant difference in the expression of caveolin-1, whereas late stages (IIIA and above) showed a 3-fold and a nearly 4-fold increase in TIMP-1 and caveolin-1 expression, respectively. These results indicate that TIMP-1 and caveolin-1 are upregulated in chronic liver diseases and that their expression correlates with the progression of HCC.

### Three-Dimensional Matrix Stiffness Recapitulates TIMP-1 and Caveolin-1 Overexpression in Hepatic Stellate Cells

Hepatic stellate cells (HSCs) are involved in wound healing in response to liver injury but can become dysregulated in HCC and promote desmoplasia and tumorigenesis.^5,6^ Given their central role in fibrosis, cancer development, and matrix remodeling, HSCs are an ideal model cell to investigate the upregulation of TIMP-1 and caveolin-1 in liver disease. Since we have shown both proteins are upregulated in cirrhosis and HCC, conditions associated with an increase in matrix stiffness, we decided to investigate their expression in HSCs cultured in biomimetic 3D matrices with tunable stiffness.

Self-assembling polypeptide matrices are a family of biomaterials that rely on short, well-defined amino acid sequences to form a hydrogel network. The short peptides self-assemble into supramolecular structures via predictable interactions, forming a fibrillar network with excellent biocompatibility, tunable physicochemical properties, and high reproducibility.^55,56^ This mechanism of *in situ* fabrication is also compatible with cell encapsulation, enabling the formation of 3D cultures with cells directly embedded in the matrix.^57^

**Figure 6:**
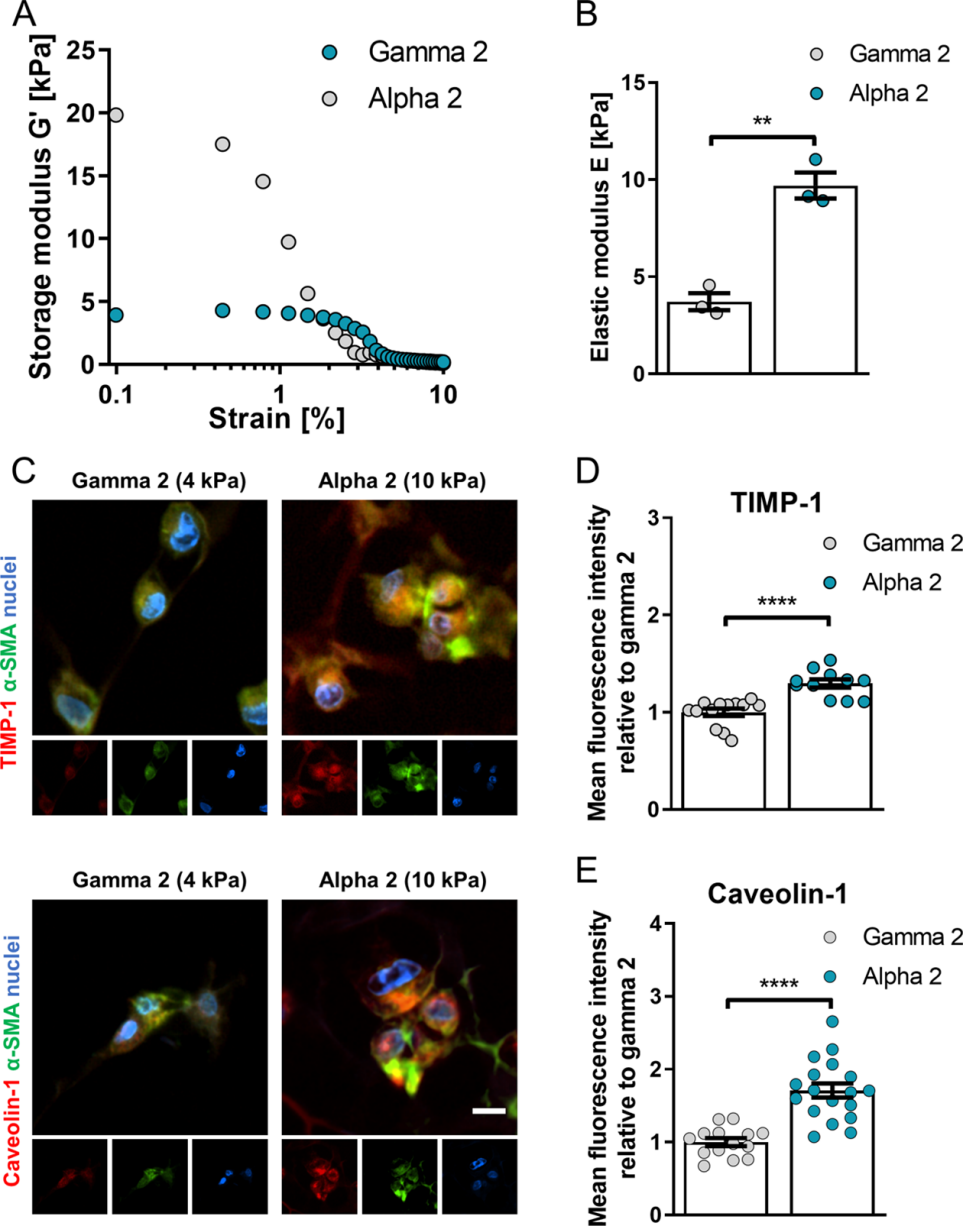
3D self-assembling peptide gels of high fibrosis-mimicking stiffness trigger upregulation of caveolin-1 and TIMP-1. (A) Average storage modulus (*G*′) across 0.1–10% strain sweep and (B) elastic modulus of PeptiGels calculated as *E* = 2 × *G*′ (1 + υ) where, υ = Poisson’s ratio of 0.5. Histogram bars represent mean ± s.e.m; dots represent individual data points. (C) Representative epifluorescent images of hepatic stellate cells caveolin-1 and TIMP-1 immunostaining of formalin fixed, paraffin embedded soft (4 kPa, gamma 2), and stiff (10 kPa, alpha 2) 3D MBG PeptiGels; protein of interest (red), alpha smooth muscle actin (green) and nuclei (blue), scale bar is 20 μm. TIMP-1 (D) and caveolin-1 (E) immunofluorescence staining for panel (C). TIMP-1 *n* = 13 and 11, caveolin-1 *n* = 14 and 18 for gamma 2 and alpha 2 respectively. Mean ± s.e.m. Markers denote significant difference between gamma 2 and alpha 2 by *t* test, ** 0.001 < *p* < 0.01, **** *p* < 0.0001.

We selected two commercially available polypeptide hydrogels of different stiffnesses, gamma 2 and alpha 2 PeptiGels (Manchester BIOGEL). We then measured their mechanical properties using oscillatory rheometry under a frequency sweep (0.1–10% strain) as well as their elastic modulus (Figure 6A,B) and confirmed that their stiffness is similar to the nominal values reported by the manufacturer for both gamma 2 (∼4 kPa) and alpha 2 (∼10 kPa), which mimic the stiffness of healthy tissue and liver fibrosis, respectively.

To elucidate the role of substrate stiffness in the regulation of TIMP-1 and caveolin-1, we encapsulated HSCs in alpha 2 and gamma 2 hydrogels and analyzed their expression *via* immunofluorescence. We observed a ∼30% increase in TIMP-1 expression and a ∼70% increase in caveolin-1 expression in stiff gels (alpha 2, 10 kPa) compared to cells on soft gels (gamma 2, 4 kPa) (Figure 6C–E), consistent with our previous results on the expression of TIMP-1 and caveolin-1 in cirrhosis and HCC. Likewise, β1 integrin blocking impaired the stiffness-dependent upregulation of TIMP-1 in HSCs cultured in 3D matrices (Figure S7). These results indicate that the HSCs cultured on 3D matrices of tunable stiffness can recapitulate the upregulation of TIMP-1 and caveolin-1 observed in cirrhosis and HCC and establish matrix rigidity as a key regulator of vesicular transport (caveolin-1) and matrix remodeling (TIMP-1) in HSCs mediated by changes in plasma membrane tension.

**Figure 7:**
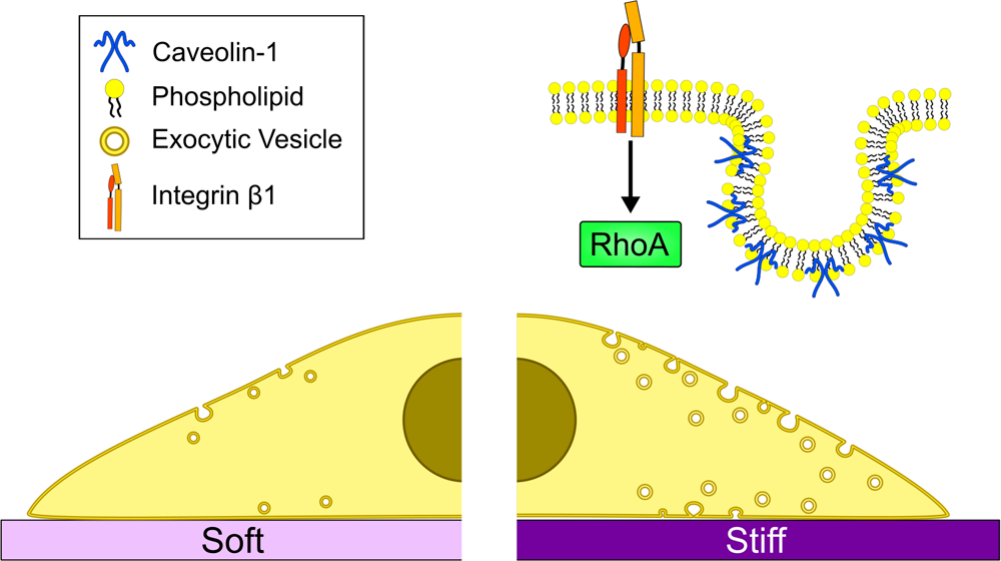
Stiffness-dependent regulation of exocytosis. High substrate stiffness triggers a mechanosignaling cascade *via* β1 integrin and RhoA that increases plasma membrane tension. In response, both caveolae formation and vesicle trafficking increase in order to buffer the high membrane tension. This, in turn, promotes the exocytosis of TIMP-1, preventing ECM degradation and maintaining the stiff environment.

The plasma membrane is a complex biological structure that can sense and adapt to changes in tension, osmotic pressure, and external forces such as shear and compression.^20^ The mechanisms of membrane tension regulation remain underexplored but have gathered interest due its emerging roles in vesicle trafficking, mechanosensing, motility, and division.^23,58^ Recently, the development of membrane tension probes, such as the FLIPPER-TR used here, has enabled the characterization of the mechanical properties of the plasma membrane and membrane-enclosed organelles,^36,59,60^ providing insight into the role of membrane tension and adaptation in vesicle trafficking^61^ and organelle function.^62^ In this study, we demonstrate that plasma membrane tension in HSCs can be regulated by matrix stiffness *via* β1 integrin and RhoA-dependent mechanosensing. The increase in membrane tension, in turn, promotes the formation of caveolae and the secretion of TIMP-1 *via* caveolin-1 and dynamin-2 dependent exocytosis (Figure 7).

Previous reports have shown that vesicular trafficking is an important modulator of membrane tension and that exocytosis upregulation leads to a reduction in this tension by adding area to the membrane^21^ and forcing water to leave the cells.^63^ Our results indicate that membrane tension in cells increases with matrix stiffness, suggesting the increase in exocytosis rate may be a negative feedback mechanism to maintain membrane tension homeostasis. Besides vesicular trafficking, cells employ other strategies to regulate membrane tension, primarily by modulating the actin cytoskeleton and cell shape.^63^ Actin contractility, known to be induced by matrix stiffness,^64^ opposes actin protrusion at the cell cortex and prevents further generation of tension by membrane extension.^34^ While more work is needed to elucidate how matrix stiffness controls membrane tension, our work provides a link between mechanotransduction and intracellular vesicular transport, which could play a role in the regulation of cell behavior by mechanosensing in both health and disease.

Here, we found that β1 integrin, but not β3, is required for the regulation of plasma membrane tension by substrate stiffness. The role of β1 integrin and RhoA in mechanosensing, contractility, and stress fiber formation is well established.^44,65^ In turn, stress fiber assembly has been found to promote caveolae flattening, either directly or indirectly by increasing membrane tension.^25^ We also found that β1 integrin is required for the modulation of vesicular trafficking in response to osmotic pressure independently from substrate stiffness. Interestingly, rapid caveolae flattening in response to osmotic swelling appears to be actin-independent,^66^ which suggests that different mechanisms govern membrane tension adaptation over short and long time-scales. β1 integrin has been proposed as an osmosensor, which can be activated in response to changes in cell volume and hypotonic stress.^67−69^ It is therefore possible that β1 integrin plays a dual role in regulating plasma membrane trafficking over different time-scales and through different mechanical cues. In future work, studying the role of other cell/ECM adhesion proteins, such as syndecan-4,^70^ could provide further insight into the mechanical regulation of membrane tension.

The regulation of membrane trafficking we observed here relies on caveolin-1, a scaffolding protein associated with both endocytic budding and exocytosis.^24^ The role of caveolin-1 in cancer remains controversial; it is associated with the regulation of vesicular transport in endocytosis, exocytosis, and transcytosis,^51,71^ acting as either an oncogene or a tumor suppressor depending on the type of tumor and its stage.^72^ The flattening of caveolae in response to high membrane tension is well documented,^19,21^ and caveolin-1 may also contribute to tension buffering through exocytosis by facilitating budding from the Golgi apparatus as well as fusion at the plasma membrane. Further evidence suggests that caveolae and caveolin-1 may participate in mechanotransduction: caveolae domains recruit and regulate RhoGTPases such as RhoA,^73,74^ mechanically gated ion channels,^75,76^ and receptors such as Akt and ERK,^77,78^ and caveolar components such as EHD2 have been shown to translocate to the nucleus and act as transcription factors upon caveolae flattening and disassembly.^79^ This emerging role of caveolae as mechanotransduction structures suggests that the link between mechanosensing and membrane tension regulation may be more complex than previously thought.

While the role of caveolin-1 in membrane tension adaptation is well established, we found dynamin-2 to be likewise indispensable for the mechano-adaptive response, particularly for the increase in exocytosis rate in response to osmotic pressure. Dynamin-2 has been classically associated with endocytosis, but it also mediates kiss-and-run exocytosis,^54^ which may enable rapid membrane expansion in response to high tension. Its function in vesicle budding from the TGN may also facilitate phospholipid shuttling to the plasma membrane to buffer tension.^80^ Moreover, dynamin-2 can be regulated by the β1 integrin/RhoA axis to modulate membrane area *via* endocytosis,^45^ which highlights how mechanosignaling pathways may intersect and regulate multiple vesicular transport mechanisms. This in turn illustrates the need to consider how other proteins involved in vesicle formation and trafficking may participate in the regulation of membrane tension. For instance, the activity of clathrin, another scaffolding protein typically associated with endocytosis, is sensitive to substrate rigidity in fibroblasts, while other endocytic pathways are independent.^50^ Clathrin may contribute to exocytosis and protein secretion by facilitating the budding of vesicles from the trans Golgi network^81^ and has been shown to form mechanosensitive signaling plaques^82^ connected to actin dynamics.^83^

While we previously reported a stiffness-dependent regulation of TIMP-1,^16^ this work sheds light on the dysregulation of ECM remodeling in HSCs. We identify a mechanism by which high matrix stiffness can upregulate TIMP-1 exocytosis, preventing its own degradation^10^ and producing a positive feedback loop that maintains the fibrotic microenvironment in cirrhosis and HCC. Notably, the membrane tension-dependent regulation of vesicular transport is likely to affect the secretion rate of other proteins and cytokines, promoting paracrine and autocrine signaling^84^ in fibrosis, inflammation, and cancer. An increasing body of evidence indicates that mechanical stimuli such as ECM stiffness are key drivers of disease in a variety of organs, including the liver, the cardiovascular system, and the lungs,^85^ which has motivated the development of therapies that aim at remodeling the microenvironment. Our groups recently showed that tamoxifen and ATRA (all-trans retinoic acid) can induce mechanical quiescence in cancer-associated fibroblasts by modulating mechanosensing and contractility.^14,64,86−88^ In this context, our work offers insights into the mechanisms that foster the development and maintenance of fibrosis and cancer as well as opportunities to modulate ECM remodeling by targeting membrane tension and vesicular trafficking.

## Conclusion

In this study, we identify a mechanism through which high substrate stiffness promotes vesicle trafficking and exocytosis by upregulating membrane tension *via* β1 integrin/RhoA-dependent mechanosensing. In response to the increase in tension, caveolae formation and vesicle trafficking act to buffer the membrane, driving the exocytosis of TIMP-1 in a caveolin-1-dependent manner. This expands on our previous findings regarding the stiffness-dependent regulation of TIMP-1 expression in HSCs^16^ and presents plasma membrane tension as a key mediator between mechanosignaling and exocytosis.

This work highlights the importance of the plasma membrane tension and its intersection with mechanotransduction. We have demonstrated that the upregulation of caveolin-1, a key element in the regulation of membrane tension, is relevant in cirrhosis and hepatocellular carcinoma, and can be recapitulated in fibrosis-mimicking 3D constructs. In light of these results, modulating plasma membrane tension could become an important therapeutic strategy in the treatment of chronic liver disease.

## Methods

### 2D Cell Culture

Primary culture-activated human hepatic stellate cells (passage 3–6, HHStec no. 5300-ScienCell) were cultured at 37 °C, 5% CO_2_ in culture medium containing DMEM/Nutrient Mixture F-12 Ham (D8437, Sigma), 10% FBS (10270–106, Gibco), 1% penicillin/streptomycin (Gibco), and 1% fungizone (15290-026, Gibco). For the AFM and TIRF microscopy experiments, HSCs were detached from culture flasks with trypsin and seeded on fibronectin coated (10 μg mL^–1^; Sigma) 35 mm with 14 mm glass bottom dishes (P35G-1.5-14-C, MatTek) or fibronectin-coated polyacrylamide gels with different rigidities and transfected. For the isotonic and hypotonic media experiments, standard culture medium (DMEM 10%FBS) was used as the former and 0.5× DMEM 10% FBS in distilled water was used as the latter (150 mOsm). For TIRF plasma membrane visualization, cells were transfected with CellLight Plasma Membrane-RFP, BacMam 2.0 (C10608, ThermoFisher) according to the standard manufacturer’s protocol. For the plasmid requiring experiments, cells were transfected with 2 μg of TIMP-1-GFP (RG201548, OriGene) cDNA using Lipofectamine 3000 (L3000001, Thermo Fisher Scientific) according to the manufacturer’s protocol. For the gene-silencing experiments, cells were transfected with 7.5 pmol of scramble control siRNA-A (sc-37007, Santa Cruz Biotechnology), caveolin-1 siRNA (sc-29241, Santa Cruz Biotechnology), and dynamin-2 siRNA (sc-35236, Santa Cruz Biotechnology) using Lipofectamine 3000 (L3000001, Thermo Fisher Scientific) according to the manufacturer’s protocol. Cell surface β1 and β3 integrins were blocked by pretreating the cell suspension for 10 min with 10 μg/mL of anti-β1 integrin (552828, BD Biosciences) or 10 μg/mL of anti-αVβ3 integrin (MAB1976Z, Millipore) blocking antibodies, respectively, prior to seeding and providing 10 μg/mL of antibodies during the course of experiment.

### Immunofluorescence

Immunofluorescent staining of liver biopsies was carried out on a formalin fixed paraffin embedded TMA-tissue microarray (cat. BC03117, US Biomax) of samples obtained from five healthy, 21 cirrhosis, 19 HCC stage I and II, 26 HCC stage III A and B, and 7 HCC stage III C and IV A patients. 3D PeptiGels embedded in agarose were dehydrated, cleared, and embedded in paraffin using the protocol described previously.^89^ PeptiGel samples were then sectioned at 5 μm using microtome. For TIMP-1, caveolin-1 and alpha smooth muscle staining TMA and hydrogel sections on glass slides were baked for 15 min at 50 °C, deparaffinized for 2 × 5 min in HistoClear (HS-200, National Diagnostics), and rehydrated in decreasing concentrations of ethanol in water (99%, 80%, 70%, and 50%) 3 min each. Antigen retrieval was performed by incubating the sections in boiling citrate buffer (pH 6) for 45 min and cooled to room temperature for 25 min. TIMP-1 antibody (MA5-13688, Thermo Fisher Scientific, 1/100), caveolin-1 antibody (15895439, BD Bioscience, 1/100), alpha smooth muscle actin antibody (ab5694abcam, 1/100), secondary goat antirabbit AlexaFluor 488 (A11034, Thermo Fisher Scientific, 1/200), and goat antimouse AlexaFluor 546 (A11030 Thermo Fisher Scientific, 1/200, 1/200) were used following the abcam TBS based staining protocol (https://www.abcam.com/protocols/immunostaining-paraffin-frozen-free-floating-protocol). Finally, coverslips were mounted in mounting reagent with 4,6-diamidino-2-phenylindole (Invitrogen, P36931). Widefield fluorescent images were taken with a Nikon Ti-e Inverted Microscope (Ti Eclipse, C-LHGFI HG Lamp, CFI Plan Fluor 40 × NA 0.6 air objective; Nikon; Neo sCMOS camera; Andor) with NIS elements AR software. Staining intensity was measured in Fiji^90^ using the “mean grey value” parameter applied to a region of interest (ROI) created for regions with positive alpha smooth muscle staining. Mean gray values for each image’s background were subtracted for each measured staining intensity.

### Polyacrylamide Substrate Fabrication

Coverslips for the TIRF and AFM experiments and glass bottom fluorodishes (P35G-1.5-14-C, MatTek) for the FLIM and confocal microscopy experiments were covered with 3-(trimethoxysilyl)propyl methacrylate (440159, Sigma) and incubated at room temperature for 5 min, wiped with a sterile, lint-free tissue, washed in dH_2_O, and left to dry at room temperature. Polyacrylamide gels of 4, 12, and 25 kPa mimicking healthy and fibrotic liver stiffnesses^35^ were prepared according to the protocol adapted from Wen *et al..*(91) Gel stiffness was varied by adding 29:1 acrylamide/bis-acrylamide to a final concentration ranging from 4.7 to 10%. A working solution of PBS, acrylamide/bis*-*acrylamide (29:1) 40% vol (A7802, Sigma), TEMED (T9281, Sigma), and 10% ammonium persulfate was mixed at concentrations to achieve varying levels of gel stiffness. A small drop of this working solution was applied to activated coverslips which were placed face down on hydrophobic, dichlorodimethylsilane (440272, Sigma)-treated glass microscope slides and left to polymerize at room temperature for 45 min. Gel-coated coverslips were removed and stored in PBS at 4 °C.

To allow polyacrylamide gels to be coated with ECM proteins, gels were functionalized to promote NHS groups. For functionalization, polyacrylamide gels were washed with PBS and coated with 50 μL of Sulfo-SANPAH (sulfosuccinimidyl 6-(4′-azido-2′-nitrophenylamino)hexanoate) (803332, Sigma) (5 mg/mL, PBS) solution per coverslip and activated with UV light for 10 min. Polyacrylamide gels were washed with PBS, coated with human plasma fibronectin (F8095, Sigma) (10 μg/mL, PBS), and incubated for 1.5 h at room temperature. Gels were washed once with PBS and cells were seeded on gels in culture medium.

### Quantitative PCR

Total RNA was extracted from cells cultured on polyacrylamide gels for 24 h with an RNeasy Mini Kit (74104, Qiagen). The RNA template was reverse transcribed into cDNA using a High-Capacity RNA-to-cDNA kit (4387406, Thermo-Fisher) according to the manufacturer’s instructions. Quantitative real-time PCR was performed on a StepOne Plus Real-Time PCR system (Applied Biosystems) using SYBR Green PCR Master Mix(4309155, Thermo-Fisher). The relative gene expression was analyzed by a comparative 2-ΔΔCt method. Primer sequences were as followed: TIMP-1 (F) TCAACCAGACCACCTTATACCA, (R) ATCCGCAGACACTCCAT; GAPDH (F) ACAGTTGCCATGTAGACC, (R) TTTTTGGTTGAGCACAGG; CAV-1 (F) CCAAGGAGATCGACCTGGTCAA, (R) GCCGTCAAAACTGTGTGTCCCT.

### Focused Ion Beam Scanning Electron Microscopy

Sample preparation and microscopy were based on previously published methods.^92^ Sample fixation of cells on polyacrylamide on glass coverslips or fibronectin coated glass coverslips was performed at room temperature for 15 min using a 4% v/v formaldehyde (Sigma, BioReagent, ≥ 36.0%) with 0.2% v/v glutaraldehyde (Electron Microscopy Sciences) solution in PBS. Washing the samples three times with cacodilate buffer (Electron Microscopy Sciences) was followed with osmication with osmium tetroxide in 2% w/v cacodilate buffer for 30 min. Then samples were washed five times with deionized water and dehydrated through a graded ethanol (Sigma, ACS reagent 99.5%) series. Samples were incubated in a diluted series of ethanol–Epon Resin (Electron Microscopy Sciences) at a 3:1, 2:1, and 1:1 ratio for 1 h each and then overnight at a 1:2 ratio. After overnight incubation with pure resin, the maximum amount of resin was removed and the samples were immediately placed in an oven at 60 °C and left to cure overnight. Then samples were placed on a SEM aluminum sample holder with carbon tape, and silver paint was applied to the surrounding area of the sample to maximize conductivity. Afterward, sputter coater (QuorumTechnologies model K575X) was used to coat the samples with 5 nm layer of chromium. Following the coating procedure, samples were introduced into an SEM/focused ion beam (Carl Zeiss, Auriga) with gallium ion beam operated at 30 kV. A region over the cells with approximately 15 × 5 × 2 μm (length × height × depth) was milled using 4 nA current. After that, the region exposed by the first milling was polished with 240 pA current and imaged by a backscattering detector with the electron beam operating at 1.5 V. The obtained data set was reconstructed using MATLAB to obtain high-resolution micrographs. The number of invaginations per length of the membrane cross-section was calculated using the ImageJ software.

### Atomic Force Microscopy

For AFM study, cell seeded polyacrylamide gels on coverslips were lifted from 24-well plates prior to measurement and immediately attached to a Petri dish with a droplet of cyanoacrylate adhesive, applied with a 10 μL pipet tip. After coverslip attachment (∼1 min), 100 μL of culture medium (DMEM 10% FBS) was applied to the coverslip in order for the AFM measurements of cells to be conducted as soon as possible (<1 h). Measurements of HSCs on polyacrylamide gels were conducted on a JPK Nanowizard-1 (JPK instruments) AFM operating in force spectroscopy mode, mounted upon an inverted optical microscope (IX-81, Olympus). AFM pyramidal cantilevers (MLCT, Bruker) with a spring constant of 0.03 N/m were used. Before conducting measurements, cantilever sensitivity was calculated by measuring the force–distance slope in the AFM software on an empty Petri dish region. For cell indentation measurements the cantilever was aligned over a central region of a cell using a 20× objective and the optical microscope. For each cell 3–5 force curves were acquired at an approach speed of 2 μm/s and a maximum set point of 0.1 V to ensure that the cantilever only probed the cell membrane. The force–distance curves were used to calculate elastic moduli in the AFM software through the application of the Hertz contact model.^93^

### Fluorescence Lifetime Imaging Microscopy

HSC cells were plated on the polyacrylamide-coated fluorodishes 24 h prior to the experiment. Culture media-diluted 2.0 μg/mL Rho Inhibitor I (CT04-A, Cytoskeleton) or 5 μL/ml RhoA Activator I (CN01-A, Cytoskeleton) were added to the cells on the gels for 3 h or 15 min, respectively, before the measurements. In order to fluorescently tag the cell membrane for the fluorescence lifetime imaging (FLIM) 1.5 μM FLIPPER-TR (cy-sc020, Cytoskeleton) was added to the cells 5 min before the experiment.

Microscopy was carried out using a Leica SP5 multiphoton inverted microscope (FLIM PMT detector and Becker & Hickl SPC-830 constant fraction discriminator) with a 488 nm pulsed laser for excitation, and photons were collected through a 600/50 nm bandpass filter. To extract lifetime information, accumulated 1 min acquisition high photon count histograms for single cells were fitted with a double exponential. Two decay times, τ1 and τ2, were extracted. The longest lifetime with the higher fit amplitude τ1 was used to report membrane tension. A longer lifetime means more tension in the membrane.^36^ Data were analyzed using FLIMFIT software.^94^

### Total Internal Reflection Fluorescence Microscopy

For total internal reflection fluorescence (TIRF) microscopy, cells were cultured and transfected in glass bottom Petri dishes with or without polyacrylamide gels to ensure high resolution and signal-to-noise. Prior to measurements, cell culture medium was changed for clear cell medium to reduce autofluorescence from the cell medium. Images of transfected cells were obtained with an inverted microscope (Eclipse Ti; Nikon) operating in TIRF mode, under ambient temperature conditions of 37 °C. Multiwavelength time lapse TIRF imaging was performed with a 63× oil immersion objective (1.3 NA, Nikon), a 488 nm diode laser, and a 561 nm diode laser for excitation coupled with emission filters 525/50 nm and 600/50 nm, respectively. Time lapse images were recorded at 0.1 Hz using a sCMOS camera (Neo, Andor) combined with the NIS elements (Nikon) control software to facilitate two color imaging across multiple regions. To minimize drift in the focus across time and multiple regions, the perfect focus system (Nikon) was used to maintain axial focus. Measurements of hypotonic conditions followed the above imaging protocol directly after the culture medium was changed to hypotonic medium.

TIRF image sequences were analyzed in ImageJ using the bleaching correction plugin to allow visualization of vesicles throughout the sequence. The coloc2 plugin was used to visualize caveolin-1 and TIMP-1 containing vesicles for analysis and vesicles were tracked using TrackMate plugin^95^ from formation to disappearance from the focal plane and, thus, emission into the surrounding medium. The kymography plugin was used to create the kymographs.

### Tissue Microarrays

Liver tissues microarrays were obtained from US Biomax, Inc., Derwood, MD (catalogue number BC03117), containing 48 cases of hepatocellular carcinoma, 5 liver cholangiocellular carcinoma, 22 liver cirrhosis, and 5 normal hepatic tissue, single core per case. Tissue samples were fixed in 10% neutral formalin for 24 h, dehydrated with gradient ethanol, cleared with xylene, and embedded in paraffin. Afterward, each slide was tested by Biomax for immunohistochemistry on one antibody specific to the tissue used in the array. 1.5 mm in diameter cores were composed into a microarray, cut into 5 μm sections by the company and baked for 30 min at 55 °C before immunostaining.

### 3D Cell Culture

Hepatic stellate cells were cultured and expanded as described for 2D cultures. After reaching 70%, confluency cells were harvested, centrifuged, and resuspended in fresh cell culture media. Twenty microliters of cell suspension was mixed with prewarmed (37 °C for 30 min) hydrogels gamma 2 and alpha 2 (PeptiGels, Manchester BIOGEL) (100 μL of each, final cell concentration 2 × 10^6^/mL). Subsequently, the gels were pipetted onto a prewetted 6.5 mm, 3.0 μm pore Transwell polycarbonate membrane insert (3415, Corning) and incubated in cell culture media for 1 h to set the gels. Following initial incubation, the samples were cultured for 7 days with media replaced every 24 h. At the end of the experiment, gels were fixed in 4% paraformaldehyde for 30 min at room temperature, washed in PBS, and embedded in 2% low melting point agarose (cat. 16520050, Thermo Fisher Scientific) in PBS.

Gel viscoelasticity analysis was performed on unfixed, acellular gels prepared using the protocol for cell-containing gels. Sample measurements were done at 37 °C using TA Instruments AR2000ex rheometer with a flat, stainless-steel cylinder. The elastic modulus (*E*) was calculated as *E* = 2*G*′(1 + υ) where υ = Poisson’s ratio of 0.5 and *G*′ is the average storage modulus across the 0.1–10% strain sweep.

### Statistical Analysis

All statistical analyses were conducted with Prism software (version 8, GraphPad). Data were collected from multiple repeats of different biological experiments or different patients to obtain the mean values and s.e.m. displayed throughout. P values have been obtained through *t* tests on unpaired samples with parametric tests used for data with a normal distribution. ANOVA and post hoc Dunnett’s test were used to perform multiple comparison test on normally distributed data. Significance was set at *P* < 0.05 where graphs show significance through symbols (*0.01 < *P* < 0.05; **0.001 < *P* < 0.01; ***0.0001 < *P* < 0.001; ****P < 0.0001).

## Supporting Information Available

Cytoskeleton stiffness increases in response to elevated substrate stiffness (Figure S1); Membrane tension in mouse epithelioid cell line (Figure S2); Caveolae diameter in different conditions (Figure S3); TIMP-1 GFP plasma membrane localization (Figure S4); mRNA expression of TIMP-1 assayed by RT-qPCR (Figure S5); Quantification of caveolin-1 (CAV-1) expression in HSCs (Figure S6); β1 Integrin blocking downregulates TIMP-1 on 3D self-assembling peptide gels mimicking healthy and fibrotic stiffnesses (Figure S7) (PDF)

## Notes

All data included in this manuscript is available from the authors.
