# Supplementary material for: Substrate Stiffness-Driven Membrane Tension Modulates Vesicular Trafficking via Caveolin-1
Source: ACS Nano. 2022 Mar 7;16(3):4322–37. doi: 10.1021/acsnano.1c10534 (PMC9007531; doi:10.1021/acsnano.1c10534)
Supplement: Supplementary file 1 — nn1c10534_si_001.pdf [file nn1c10534_si_001.pdf]

Substrate stiffness-driven membrane tension modulates vesicular trafficking via caveolin-1

Dariusz Lachowski, Carlos Matellan, Sahana Gopal, Ernesto Cortes, Benjamin K. Robinson, Alberto Saiani, Aline F. Miller, Molly M. Stevens and Armando E. del Río Hernández

Supplementary Figure S1. Cytoskeleton stiffness increases in response to elevated substrate stiffness.

Supplementary Figure S2. Membrane tension in mouse epithelioid cell line.

Supplementary Figure S3. Caveolae diameter in different conditions.

Supplementary Figure S4. TIMP-1 GFP plasma membrane localization.

Supplementary Figure S5. mRNA expression of TIMP-1 assayed by RT-qPCR.

Supplementary Figure S6. Quantification of Caveolin-1 (CAV-1) expression in HSCs.

Supplementary Figure S7. β1 Integrin blocking downregulates TIMP-1 on 3D self-assembling peptide gels mimicking healthy and fibrotic stiffnesses.

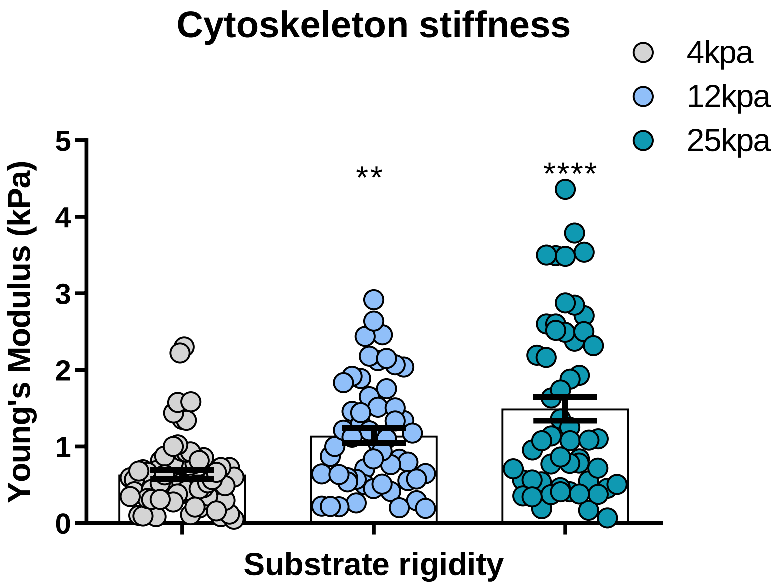

Supplementary Figure 1. Cytoskeleton stiffness increases in response to elevated substrate stiffness. Hepatic stellate cells membrane stiffness on 4, 12 and 25 kPa polyacrylamide substrates measured as Young's Modulus. Histogram bars represent mean ± s.e.m. Data are representative of 3 independent experiments and 100 cells analysed. Markers denote significant difference from 4 kPa condition by ANOVA with Dunnett's post hoc test, ** $0.001<p< 0.01$, **** $p<0.0001$.

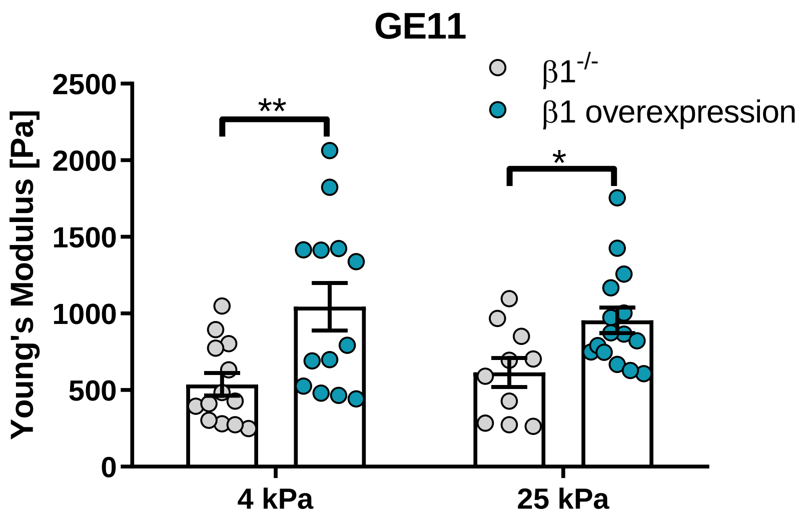

Supplementary Figure S2. Membrane tension in mouse epithelioid cell line. Membrane stiffness measured by AFM in integrin β$1^{-/-}$ and integrin β1 overexpressing mouse epithelioid cell line cultured on 4 kPa and 25 kPa polyacrylamide substrates. In both cases, β1 integrin knockdown cells present a significantly lower membrane tension compared to cells overexpressing integrin β1. Data are representative of 3 independent experiments. ** $0.001<p< 0.01$, **** $p<0.0001$.

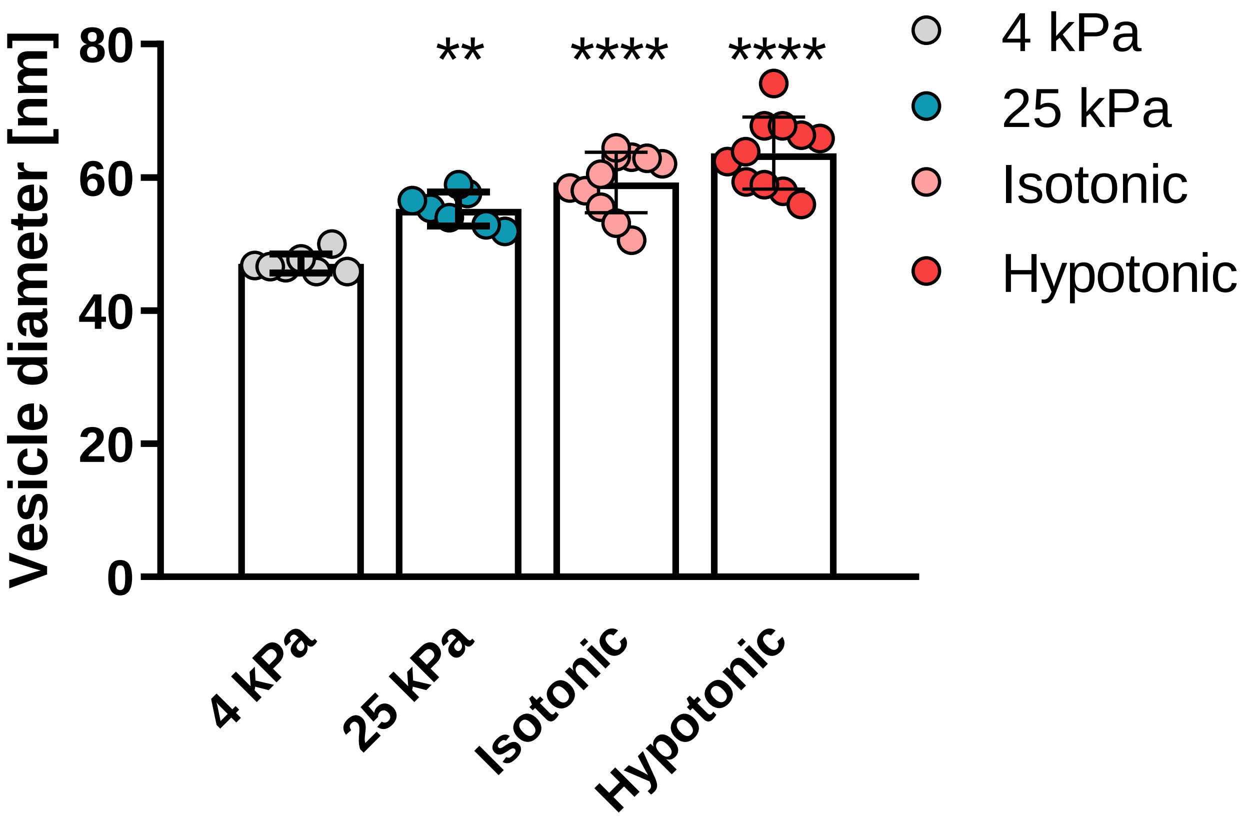

Supplementary figure S3. Caveolae diameter in different conditions. Membrane vesicle diameter the cells on 4 and 25 kPa polyacrylamide substrates, isotonic and hypotonic media represented on Figure 4 (A) and Figure 5 (A). Histogram bars represent mean ± s.e.m; dots represent individual data points. Data are representative of averages of 7 sections from 3 cells for 4 and 25 kPa each and averages of 11 sections from 3 cells for isotonic and hypotonic each. Markers denote significant difference from 4 kPa condition by ANOVA with Dunnett's post hoc test, ** $0.001<p<0.01$, **** $p<0.0001$.

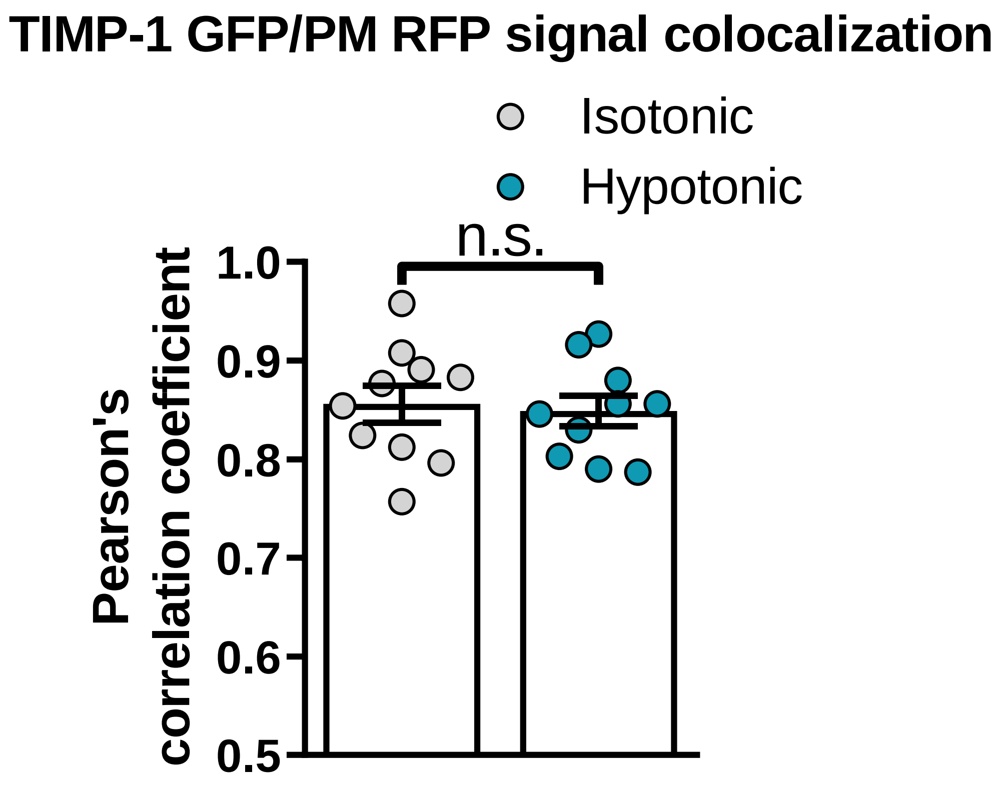

Supplementary figure S4. TIMP-1 GFP plasma membrane localization. TIMP-1 GFP and BacMam plasma membrane RFP TIRF immunofluorescence signal colocalization calculated as Pearson's correlation coefficient. Histogram bars represent mean ± s.e.m; dots represent individual data points. n.s. denotes no significant difference by t-test.

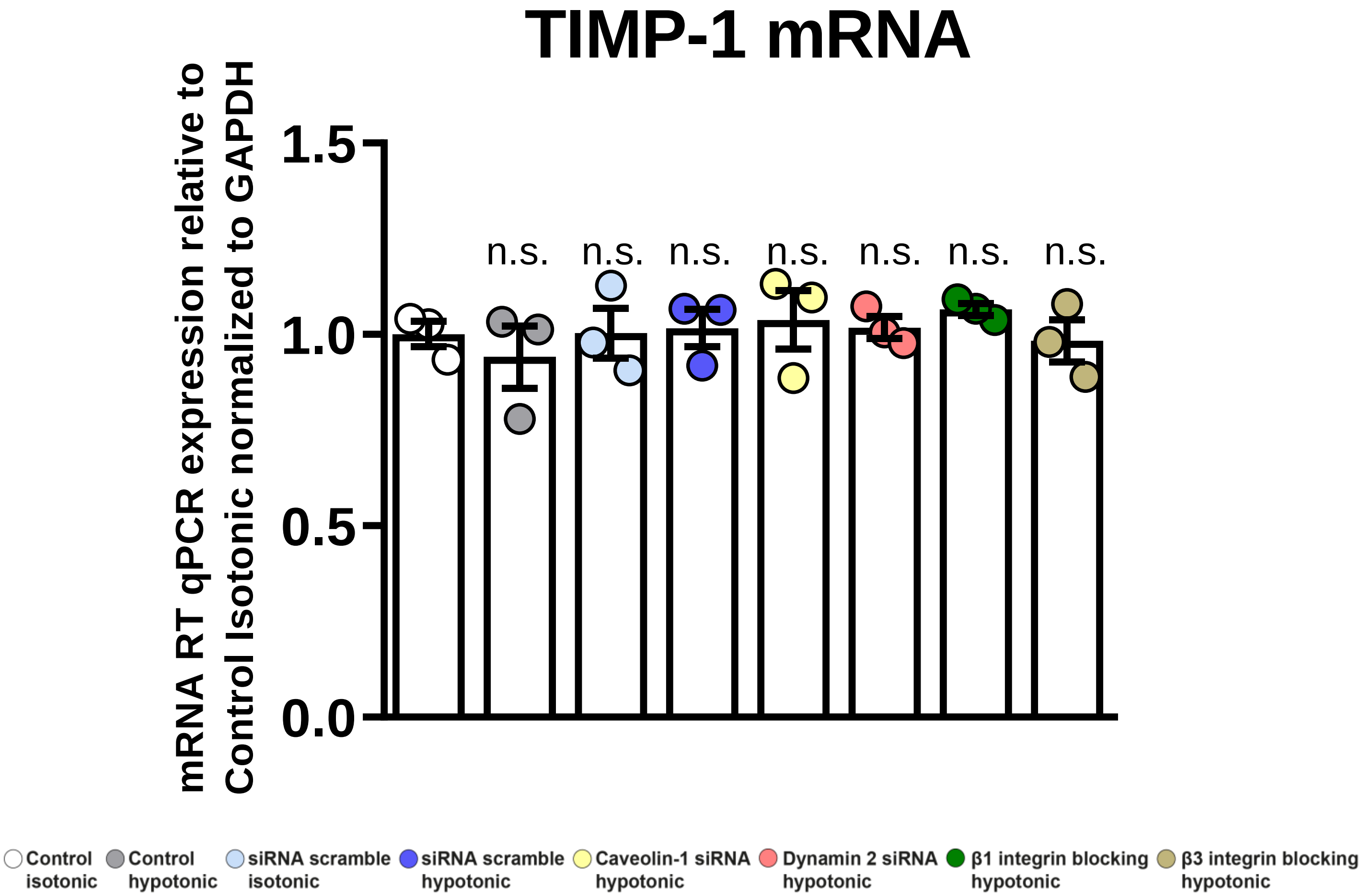

Supplementary Figure S5. mRNA expression of TIMP-1 assayed by RT-qPCR, normalised to control GAPDH relative to control isotonic condition. Histogram bars represent mean ± s.e.m; Data are representative of n = 3 experimental replicates. Markers denote significant difference from control isotonic condition by one way ANOVA with Dunnett's post hoc test, n.s. – not significant.

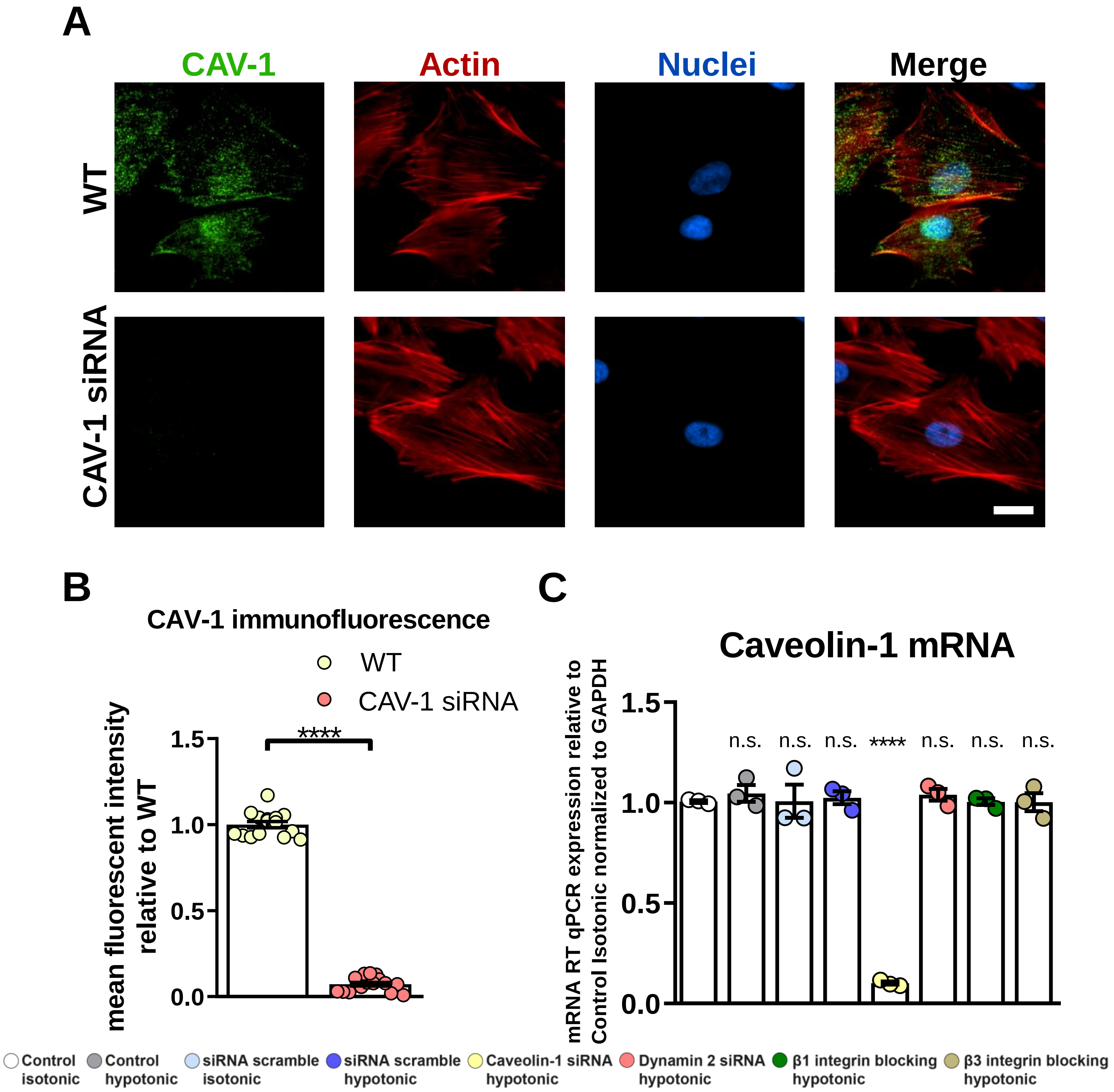

Supplementary figure S6. Quantification of Caveolin-1 (CAV-1) expression in HSCs. (A) Representative immunofluorescent images demonstrating wildtype (WT) and CAV-1 siRNA transfected hepatic stellate cells (HSCs) immunostained for CAV-1; **(B)** Quantification of mean fluorescence intensity of **(A)**. Number of cells – 15 for each condition. Scale bar represents 20 µm. Histogram bars represent mean ± s.e.m; Data are representative of n = 3 experimental replicates. **** p<0.0001 by t-test. **(C)** Caveolin-1 expression assayed by RT-qPCR, normalised to control GAPDH relative to control isotonic condition. Histogram bars represent mean ± s.e.m; dots represent individual data points. Markers denote significant difference from control isotonic condition by one way ANOVA with Dunnett's post hoc test, **** p<0.0001.

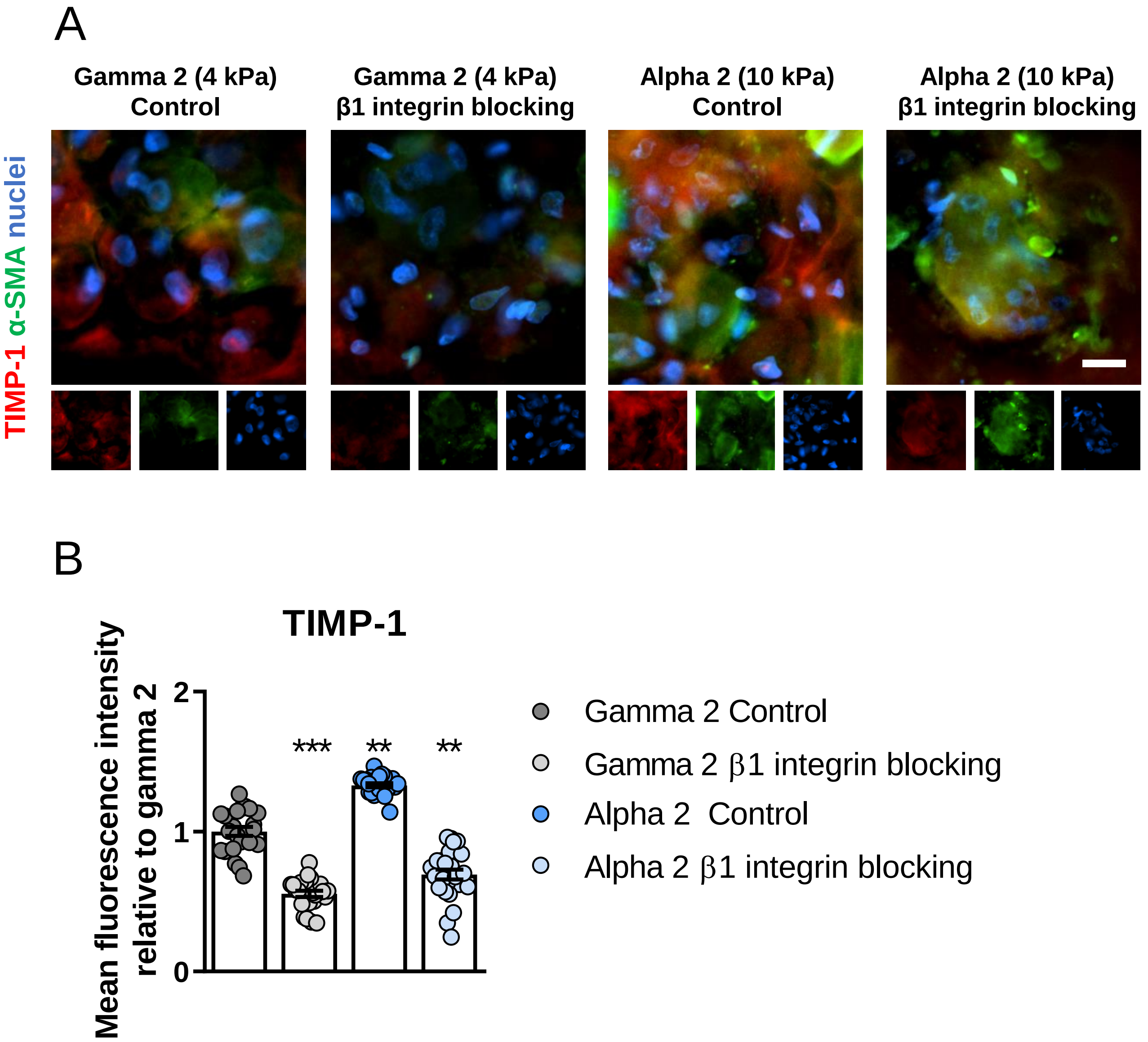

Supplementary Figure S7. β1 Integrin blocking downregulates TIMP-1 on 3D self-assembling peptide gels mimicking healthy and fibrotic stiffnesses. (A) Representative epifluorescent images of hepatic stellate cells (control – no treatment, and β1 integrin blocking) TIMP-1 immunostaining of formalin fixed, paraffin embedded soft - 4 kPa gamma 2, and stiff - 10 kPa alpha 2 3D MBG PeptiGels; TIMP-1 (red), alpha smooth muscle actin (green) and nuclei (blue), scale bar is 20 μm. n=23 ROIs for each group. Mean ± s.e.m. Markers denote significant difference from control Gamma 2 Control condition by one way ANOVA with Dunnett's post hoc test, ** $0.001<p<0.01$, *** $0.0001<p<0.001$.
